# Connexin 43‐Enriched Vesicles Improve Synchronization in hiPSC‐Derived Cardiomyocytes

**DOI:** 10.1002/advs.202521032

**Published:** 2026-04-28

**Authors:** Nima Momtahan, Anna K. McClain, Andrea Trementozzi, Sogu Sohn, Lingxia Qiao, Chi Zhao, Padmini Rangamani, Jeanne C. Stachowiak, Janet Zoldan

**Affiliations:** ^1^ Department of Biomedical Engineering The University of Texas at Austin Austin Texas USA; ^2^ Department of Pharmacology, School of Medicine University of California San Diego La Jolla California USA; ^3^ Department of Mechanical and Aerospace Engineering, Jacobs School of Engineering University of California San Diego La Jolla California USA; ^4^ The University of Texas at Austin McKetta Department of Chemical Engineering Austin Texas USA

**Keywords:** biovesicles, cardiomyocytes, hiPSCs, synchronization

## Abstract

Human induced pluripotent stem cell‐derived cardiomyocytes are valuable for studying cell–cell communication and synchronization, but remain immature and often lack robust electrical and mechanical coupling. To address this, we investigated gap junction‐mediated communication and developed plasma membrane vesicles enriched in functional connexin hemichannels, termed Connectosomes, to enhance intercellular coupling. Connectosomes display properly oriented connexins and enrich the Cx43 expression at cell–cell borders between cardiomyocytes. Through mathematical modeling and experimental validation, we demonstrate that Connectosome incorporation reinforces endogenous gap junctions, promotes synchronous calcium transients, and improves spatial coordination of beating across networks. Mechanistic studies using engineered cell lines with tagged connexin‐43 confirm that channel orientation and functionality are critical, supporting a model in which Connectosomes contribute to gap junction coupling. These results show that Connectosomes can synchronize the beating of immature cardiomyocytes by boosting electrochemical communication, laying the groundwork for future therapeutic advances.

## Graphical abstract

Plasma membrane‐derived Connectosomes enriched in connexin‐43 are produced from donor hiPSCs and applied to hiPSC‐derived cardiomyocytes (hiPSC‐CMs). Connexin‐43 channels on Connectosomes remain functional, strengthening intercellular electrochemical coupling and increasing network synchronization over time. This modular, non‐genetic approach enables rapid, scalable tuning of coupling in immature hiPSC‐CM cultures.

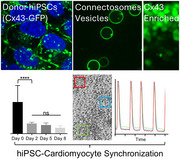

## 1 Introduction

Significant progress has been made in the cultivation of induced pluripotent stem cells (hiPSCs) towards specific lineages for tissue engineering applications and cell transplantation. However, the next big challenge is to move from cells to functional engineered tissues. Functional tissues require 3‐dimensional (3D) organization, with cells interconnected and able to efficiently communicate throughout.

One of the basic forms of such intercellular communication occurs through electrochemical coupling. Electrochemical coupling persists throughout the tissue and is essential for tissue homeostasis and coordinated growth. A key requirement for electrochemical integration is that cells be connected by a network of cell–cell junctions.

Such electrochemical coupling is specifically essential between cardiomyocytes and occurs through gap junctions. Gap junctions are composed of a hemichannel formed by six transmembrane proteins, termed connexins, that interconnect the cytosol of two adjacent cells. There are three main connexin isoforms, classified according to molecular weight, in the adult heart: connexin‐40, connexin‐43 (Cx43), and connexin‐45, with Cx43 being the most abundant [1]. Cx43 hemichannels preferentially localize to the intercalated disks [2]. These channels provide the basis for intercellular communication in the cardiovascular system, allowing the transfer of ions and small molecules between cells. Specifically, in the heart, gap junctions mediate the transfer of calcium ions, which is crucial for the orderly spread of the wave of electrical excitation responsible for synchronous contraction [3, 4]. Cx43 is, therefore, essential for cardiac function because of its key role in synchronizing cardiomyocyte (CM) beating [5, 6].

Alterations in Cx43 expression and distribution have a significant impact on conduction in the heart [7] and have been observed in myocardial diseases such as hypertrophic cardiomyopathy, heart failure, and ischemia [8]. Changes in Cx43 expression have also been associated with age‐related alterations in the heart rhythm [9]. Cx43 knockdown in mice is prenatal lethal [10], and mice with conditional Cx43 knockdown have slower ventricular conduction, higher propensity for arrhythmias, and suffer from sudden cardiac death by two months of age [6].

While the loss of Cx43 decouples CMs, the high expression of Cx43 has been correlated with cell coupling and integration [11, 12]. Specifically, overexpression of Cx43 in cells transplanted from skeletal muscle resulted in improved intercellular mechanical and electrical coupling and reduced post‐infarct arrhythmia [12]. Even non‐excitable cells, such as HEK293, with Cx43 overexpression, can be electrically coupled to CMs [11]. Yet, viral transfection, which is currently the most efficient way to overexpress a protein, has its own set of risks associated with it, specifically when transfection is performed in an in vivo setting. Building on our previously reported method for harvesting connexin‐enriched membrane vesicles [13], instead of genetically engineering cells to over‐express Cx43, we propose a biomaterial strategy using connexin‐enriched cell‐membrane‐derived vesicles to promote electrochemical coupling.

In the current study, we examine the role of Cx43‐enriched membrane‐derived vesicles in the electrochemical coupling of CMs differentiated from hiPSCs (hiPSC‐CMs). Specifically, we hypothesized that these membrane‐derived vesicles would reinforce gap junction‐mediated electrochemical coupling, coordinating action potentials and downstream calcium transients between cells and synchronizing their beating. We isolated Cx43‐enriched membrane‐derived vesicles from hiPSCs, which are inherently rich in Cx43, and investigated how the presence of functional and properly oriented connexin hemichannels affects the beating kinetics of treated hiPSC‐CMs. Our results indicate that Cx43‐rich membrane‐derived vesicles retained high amounts of functional Cx43 channels. When applied to hiPSC‐CMs, these membrane‐derived vesicles reinforced gap junctions between cardiac cells by actively participating in connexon formation, thereby improving the synchronization of hiPSC‐CM beating over long distances. While we demonstrate here the use of hiPSC‐derived Cx43‐enriched membrane‐derived vesicles to enhance the electrochemical coupling of hiPSC‐CMs, this approach could be extended to patient‐specific hiPSC sources in future work. Cardiac tissue serves as an initial example, but with further validation, the same strategy could potentially be extended to other systems, supporting the transition from individual cells to electrochemically functional tissues.

## 2 Results

### 2.1 hiPSCs Have High Levels of Functional Cx43 on Their Membranes

**FIGURE 1:**
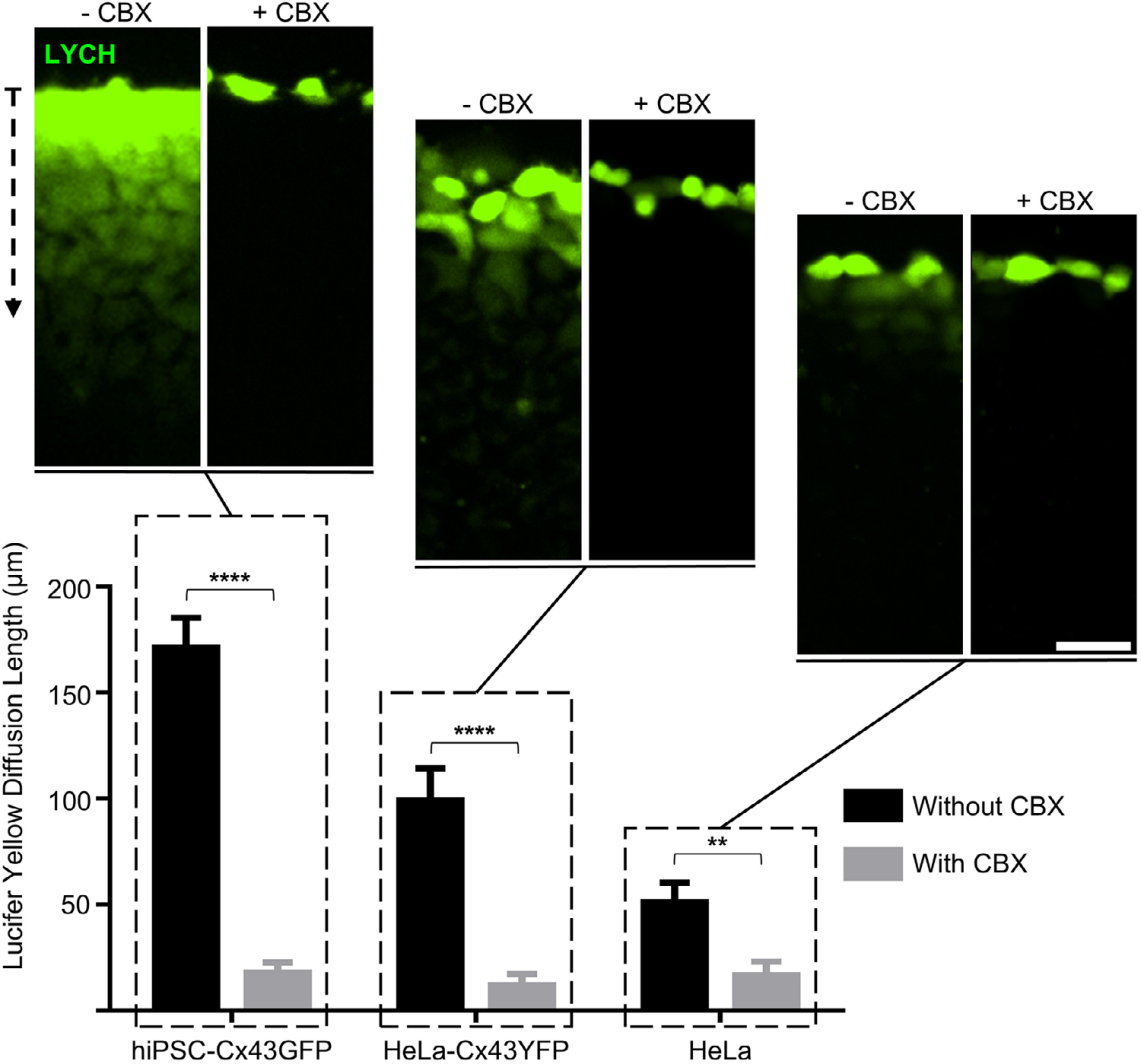
Scratch dye loading assay. Confluent monolayers of Cx43GFP reporter hiPSCs, HeLa‐Cx43YFPs, and HeLa cells were scratched to compromise the plasma cell membrane and loaded with LYCH. LYCH dye diffusion from the scratch site into neighboring cells was used as a readout of gap junction‐mediated intercellular coupling. Representative fluorescence images and quantification of LYCH diffusion length are shown. As a negative control, 100 µM CBX was added to block the gap junction. Data are mean ± SD; *n* = 3 independent experiments per condition. Two‐way ANOVA with Šidák's multiple comparisons; significance as indicated (****p < 0.0001; **p < 0.01). Scale bars: 20 µm.

In this study, we harvested Connectosomes directly from the plasma membrane of three different donor cell lines: hiPSCs expressing mEGFP‐tagged connexin‐43 (hiPSC‐Cx43GFP) and two control HeLa cell lines (wild‐type and stably transfected, inducible tet‐on HeLa cells expressing Cx43 with a C‐terminal YFP modification, HeLa‐Cx43YFP). We, therefore, first characterized Cx43 expression and gap junction network connectivity in these donor cell lines using scratch‐loading assays with lucifer yellow carbohydrazide (LYCH). LYCH is a membrane‐impermeable fluorescent dye that passes only through the gap junctions of the cells and, therefore, the length of dye diffusion is indicative of cell coupling by gap junctions [14]. Quantification of dye length scale diffusion in donor monolayers showed that hiPSC‐Cx43GFPs exhibited the greatest diffusion length (171.3 ± 14.1 µm), followed by HeLa‐Cx43YFP (99.3 ± 15.0 µm) and WT HeLa (51.3 ± 9.2 µm) (Figure 1). Because dye diffusion beyond the first cell layer with compromised membranes (induced by the scratch) depends on functional intercellular coupling through gap junctions, greater diffusion length indicates a more intact and functional gap junction network. These results suggest that hiPSCs exhibit greater gap‐junctional communication than wild‐type HeLa cells or even HeLa cells that overexpress Cx43. To directly assess Cx43 protein abundance in the donor cell lines, we performed western blotting for Cx43 using GAPDH as a loading control (Figure S1). This analysis confirmed higher total Cx43 levels in hiPSCs compared to HeLa‐Cx43YFP cells.

To further validate these results, we repeated these experiments in the presence of a gap junction blocker. Carbenoxolone (CBX) is a glycyrrhetinic acid derivative used to block intercellular communication through gap junctions [15]. It has also been reported that CBX does not have selectivity for any subtypes of connexin molecules [15]. The hiPSC Cx43GFPs, HeLa‐Cx43YFP, and wild‐type HeLa cells exposed to 100 µM of CBX significantly lowered the diffusion length of LYCH by 90 ± 3%, 88 ± 5%, and 67 ± 3% (to 17.8 ± 4.7, 11.8 ± 5.5, and 16.7 ± 6.4 µm), respectively (Figure 1). These results indicate that dye diffusion depends on the presence of active connexin channels in the cell membrane. Having established differences in functional coupling and Cx43 enrichment among donor cell types, we next characterized the Connectosomes generated from these donor cell types.

### 2.2 Connectosomes can be Isolated from Human Induced Pluripotent Stem Cells (hiPSCs)

**FIGURE 2:**
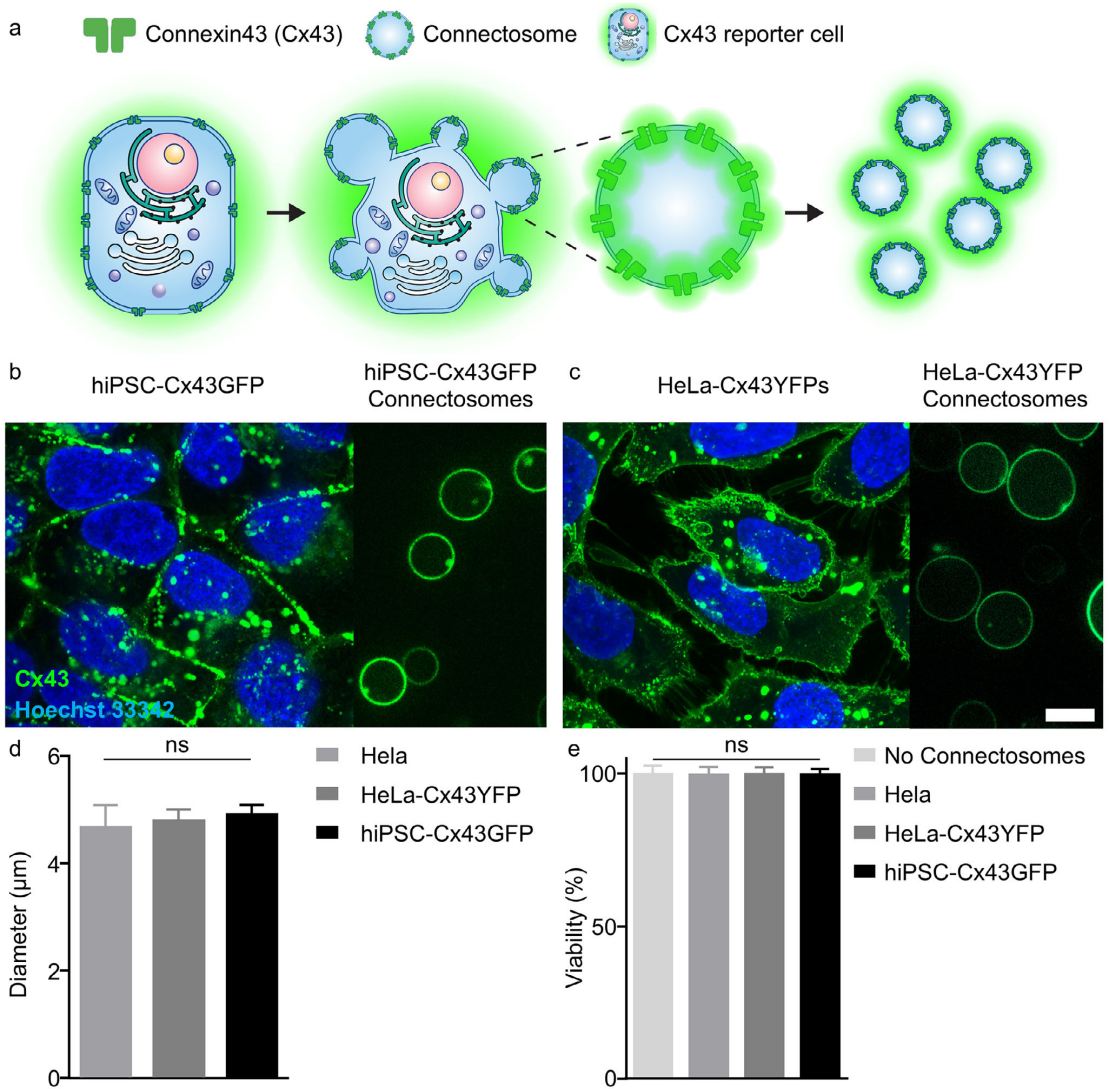
Characterization of Connectosomes. (a) Schematic view of Connectosomes generated from Cx43 reporter cells. (b,c) Representative fluorescence images of a confluent monolayer of Cx43‐GFP reporter hiPSCs (b, left) and HeLa‐Cx43YFP reporter cells (c, left), and the corresponding harvested Connectosomes from each donor type (right). Cx43 is shown in green, and nuclei in donor‐cell images are stained with Hoechst 33342 (blue). (d) Average diameter of Connectosomes harvested from hiPSCs, HeLa‐Cx43YFPs, and wild‐type HeLa cells. (e) Connectosome cytotoxicity assessed by measuring hiPSC viability 24 h after exposure to Connectosomes harvested from the indicated cell lines or a no‐Connectosome control. Data are mean ± SD; *n* = 4 independent experiments; one‐way ANOVA; *p*‐value = 0.9, ns, not significant for (d) and (e). Approximately 6.25 × 10^4^ Connectosomes were counted and measured for each measurement in (d). Scale bar: 5 µm (b,c).

Once donor cells were characterized, we next demonstrated that we could generate Connectosomes, which are membrane‐derived vesicles containing a high density of functional, properly oriented connexin hemichannels. We produced Connectosomes from donor cells using osmotic shock, coupled with mild chemical vesiculation that temporarily destabilizes the cortical cytoskeleton and induces plasma membrane budding (Figure 2a). Both donor HeLa‐Cx43‐YFPs and hiPSC‐Cx43‐GFPs lines exhibited strong reporter fluorescence, consistent with high Cx43 expression (endogenous in hiPSCs and overexpressed in HeLa), and because vesicles bud from these donor plasma membranes, their membrane‐protein composition, including Cx43 abundance, reflects the donor cell surface [16, 17] (Figure 2b,c). Connectosomes were harvested after 8 h, and the average diameter of the Connectosomes was about 5 µm, irrespective of donor cell type. Approximately 2.5 × 10^7^ Connectosomes were collected from each T‐75 flask at high confluency (approximately 9 × 10^6^ cells), demonstrating the potential scalability of this process.

### 2.3 Connectosomes are Biocompatible

We tested the impact of Connectosome treatment on hiPSC viability. While we have previously demonstrated that Connectosomes are not generally cytotoxic [18, 19], hiPSCs are uniquely sensitive to any perturbations, necessitating a more careful evaluation. Our data demonstrated that hiPSCs incubated for 24 h at 37°C with Connectosomes maintained high viability, and no significant difference in hiPSC viability was observed (Figure 2e) between cells exposed to Connectosomes harvested from the three different donor cell lines. Biocompatibility is further supported by the subsequent synchronization studies (Figures 5 and 6), in which hiPSC‐CMs remained viable for 14 days following treatment with Connectosomes, demonstrating sustained cell health under these conditions.

### 2.4 Cx43 Channels on Connectosomes are Functional

**FIGURE 3:**
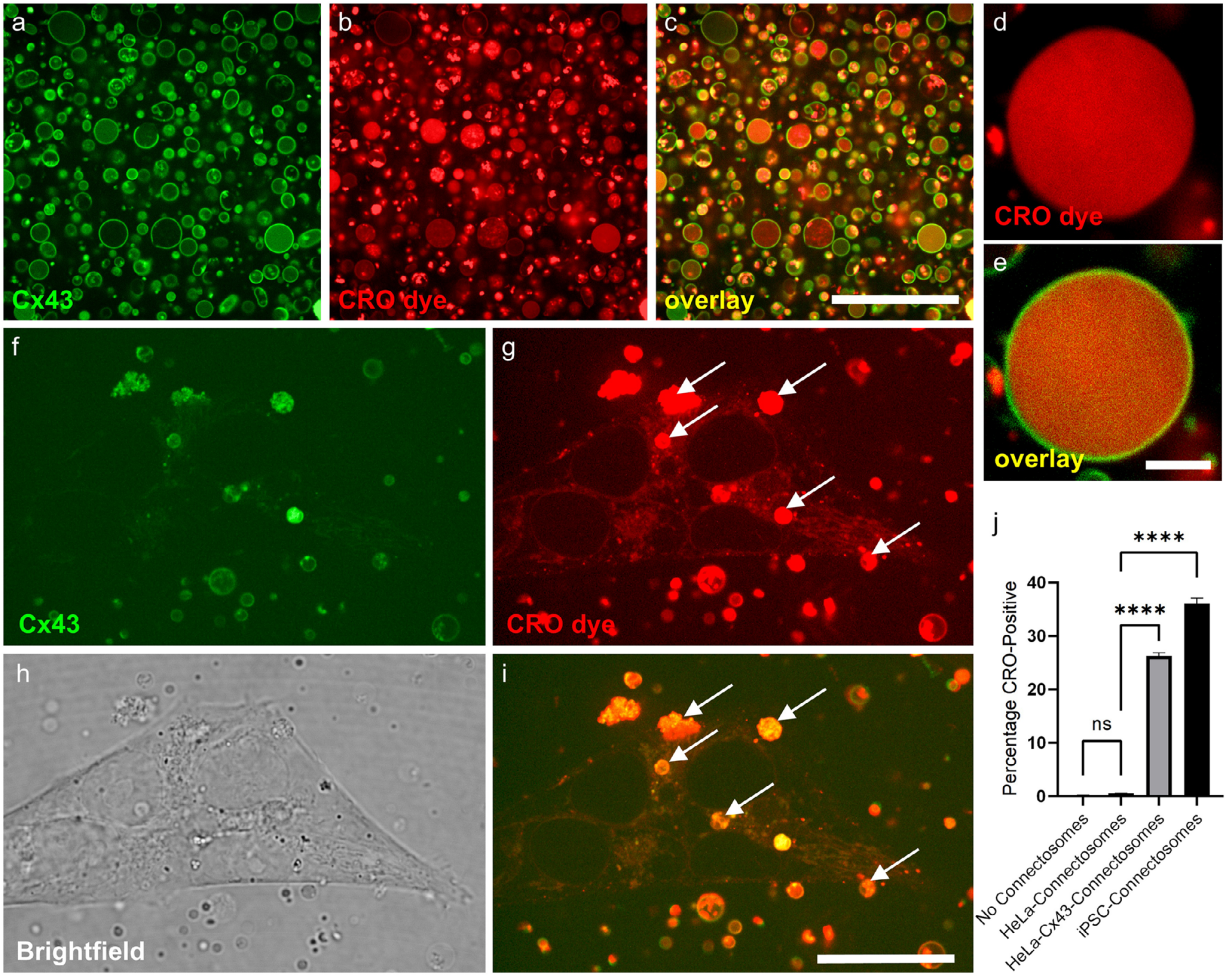
Connectosome dye loading and delivery to hiPSCs. Cx43‐GFP reporter hiPSCs were loaded with calcein red orange (CRO) dye prior to Connectosome extraction. (a–c) Isolated Connectosomes exhibit a green fluorescent membrane (Cx43‐GFP; a,e) and retain red CRO signal (b,d), with merged images shown (c,e). Higher magnification view of an individual CRO‐loaded Connectosome showing CRO fluorescence (d) and the merged overlay (e). For dye delivery, CRO‐loaded Connectosomes were added to non‐reporter hiPSCs (10:1 Connectosomes:cells) and imaged immediately. Connectosomes attached within minutes, and the CRO signal was observed diffusing into the recipient cytosol (f–i). Representative images show Cx43‐GFP (f), CRO (g), brightfield (h), and overlay (i). White arrows indicate Connectosomes attached to hiPSCs during CRO transfer. (j) Flow cytometry quantification of CRO‐positive hiPSCs 24 h after exposure to the indicated Connectosomes. Data are mean ± SD; *n* = 4 independent experiments; one‐way ANOVA with Tukey's multiple‐comparisons test; ns, not significant; ****p < 0.0001. Scale bars, 10 µm (a–c), and 1 µm (d,e).

To study the functionality of Cx43 channels on Connectosomes, we isolated dye‐loaded Connectosomes from the reporter hiPSC‐Cx43GFPs donor cells. This approach enabled visualization of Cx43‐positive Connectosome membranes and their encapsulation of the calcein red‐orange (CRO) acetomethoxy (AM) dye (Figure 3a–e). The CRO dye is membrane‐permeable. However, once it enters the cell's cytosol, it becomes membrane‐impermeable because intracellular esterases cleave the AM ester group, yielding a hydrophilic, membrane‐impermeable product.

In live cells, the gating of connexin channels is controlled by calcium ions (Ca^2^^+^), with channels remaining closed in the presence of calcium and opening in the absence of calcium [20]. Similarly, we demonstrated the functionality of the connexin channels on Connectosomes by releasing the encapsulated CRO dye through Ca^2^^+^ modulation. CRO dye was contained inside Connectosomes in the presence of Ca^2^^+^. Once Ca^2^^+^ was removed from the Connectosomes suspension, the dye was released (Figure S3a,b).

While the above experiment demonstrated the ability to control Cx43 gating on the Connectosomes membrane, we next tested the functionality of these connexin channels by performing dye delivery experiments into live cells. Non‐reporter hiPSCs were imaged immediately after the addition of CRO‐loaded Connectosomes. Connectosomes attached to these cells within minutes, and the CRO dye from the Connectosomes diffused into the cytosol of the hiPSCs (Figure 3f–i). To confirm that the red fluorescence signal was not due to cellular autofluorescence, we imaged untreated hiPSCs (no‐Connectosomes) using the same acquisition settings. Minimal signal was detected in the red and green channels (Figure S2a). We additionally quantified CRO delivery by flow cytometry by measuring the percentage of CRO‐positive hiPSCs 24 h after exposure to Connectosomes harvested from different cell types, observing near‐background CRO positivity in negative controls (no‐Connectosomes, 0.173 ± 0.030%; regular HeLa‐Connectosomes, 0.555 ± 0.039%) and a statistically significant increase with Cx43‐enriched Connectosomes (HeLa‐Cx43‐Connectosomes, 26.3 ± 1.09%; hiPSC‐Connectosomes, 36.0 ± 2.28%; Figure 3j, Figure S2b). These observations suggest that Cx43 channels on the Connectosome can engage the target membrane and enable solute transfer. Having confirmed Cx43 channels on the Connectosome are functional, we next asked whether this translates to improved functional coupling in hiPSC‐CM networks.

### 2.5 Connectosomes Synchronize the Beating of hiPSC‐CMs

**FIGURE 4:**
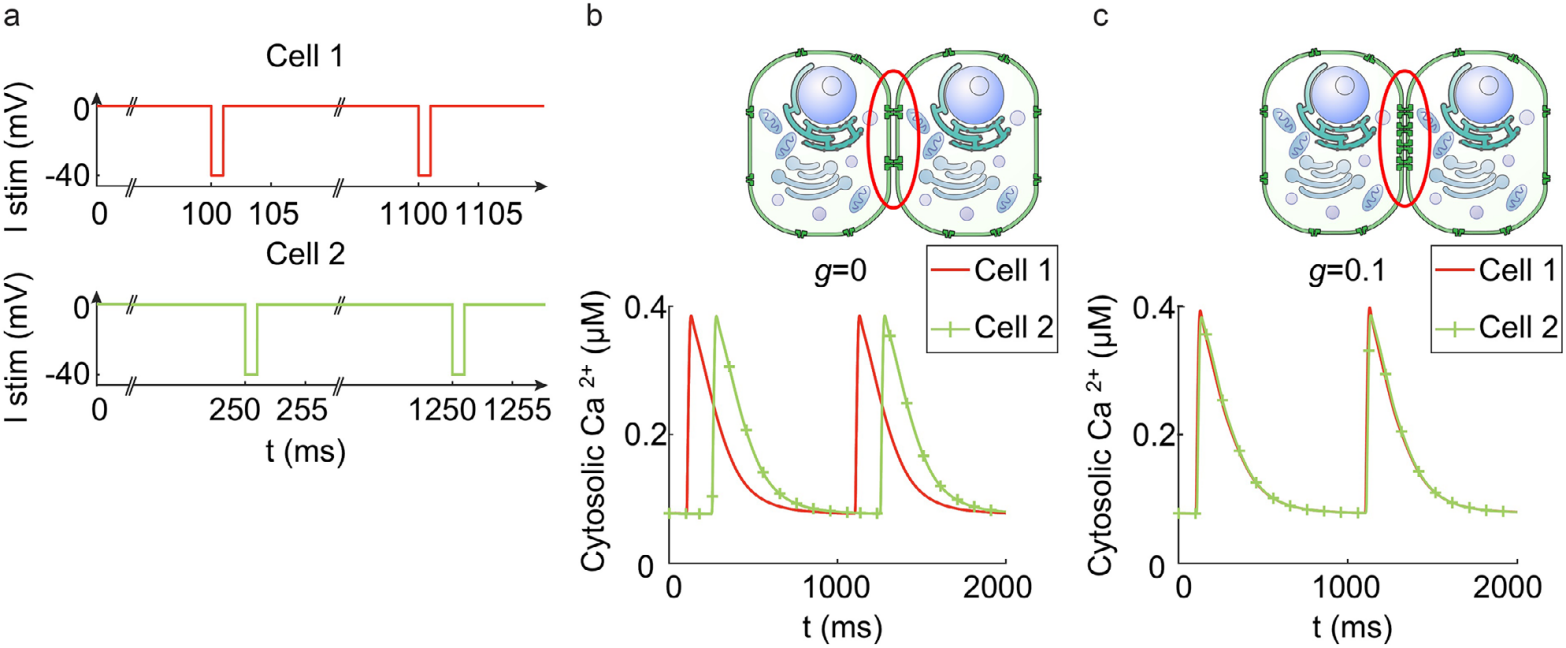
A computational model predicts that Connectosome treatment may improve the synchronicity of calcium dynamics in adjacent cells. (a) The applied stimulus current for cells 1 and 2. It has a period of 1000 ms, an amplitude of −40 mV, and a duration of 1 ms for both cells. In each period, the stimulus begins at 100 ms for cell 1 (upper panel) and at 250 ms for cell 2 (lower panel). (b) Simulated calcium dynamics in cells 1 and 2 when the gap junction conductance g is 0 (no‐Connectosome treatment). (c) Simulated calcium dynamics in cells 1 and 2 when the gap junction conductance g is 0.1 mS/µF (Connectosome treatment).

To interpret how changes in intercellular coupling could affect the synchronization dynamics of hiPSC‐CMs, we developed a minimal two‐cell model that relates effective gap‐junctional conductance to calcium‐transient timing. Based on the above experiments, which demonstrate the functionality of Cx43 channels and Connectosomes’ engagement with the cell membrane, we hypothesized that Connectosomes could increase electrochemical coupling between neighboring CMs by increasing the number of functional connexin channels available for intercellular communication. Calcium ions are key signaling molecules involved in regulating cardiac muscle contraction and coordinating electrical activity in the heart. Such coordinated function is enabled by Cx43‐based gap junctions, which interconnect cardiac cells and allow for the transfer of electrical signals and ions, including calcium [4, 5, 6]. To test the functionality of Connectosomes and their effect on cardiac beating, we first developed a computational model to predict how increasing Cx43 between 2 adjacent cells impacts the synchronicity of calcium dynamics (see Materials and Methods for details). In brief, we considered two cells and used the computational model, developed by jaeger et al. [21], to describe the dynamics of membrane potential and calcium in each cell. However, to model the gap junction, we added the terms $-g(I_{gap}^{\left(1\right)}-I_{gap}^{\left(2\right)})$ and $-g(I_{gap}^{\left(2\right)}-I_{gap}^{\left(1\right)})$ in the membrane potential equation for cells 1 and 2. Here, *g* is the gap junction conductance, and $I_{gap}^{\left(1\right)}$ and $I_{gap}^{\left(2\right)}$ represent the gap junction currents in cells 1 and 2, respectively. To reproduce the non‐synchronized calcium dynamics observed in the absence of Connectosome treatment, we chose a different starting time for the applied stimulus current: for cell 1, the current begins at 100 ms, while for cell 2, it starts at 250 ms (Figure 4a). Then, we set *g* to 0 to represent the untreated condition (Figure 4b), and increased g to simulate the Connectosome treatment case (Figure 4c). This is based on the assumption that the treatment with hiPSC‐Connectosomes may increase effective intercellular coupling by supplying additional functional connexin channels. We found that the Connectosome treatment significantly improves the synchronicity of calcium dynamics between two adjacent cells (Figure 4b,c). These simulation results indicate that the Connectosome treatment may increase the synchronicity of calcium dynamics via Cx43‐based gap junctions.

**FIGURE 5:**
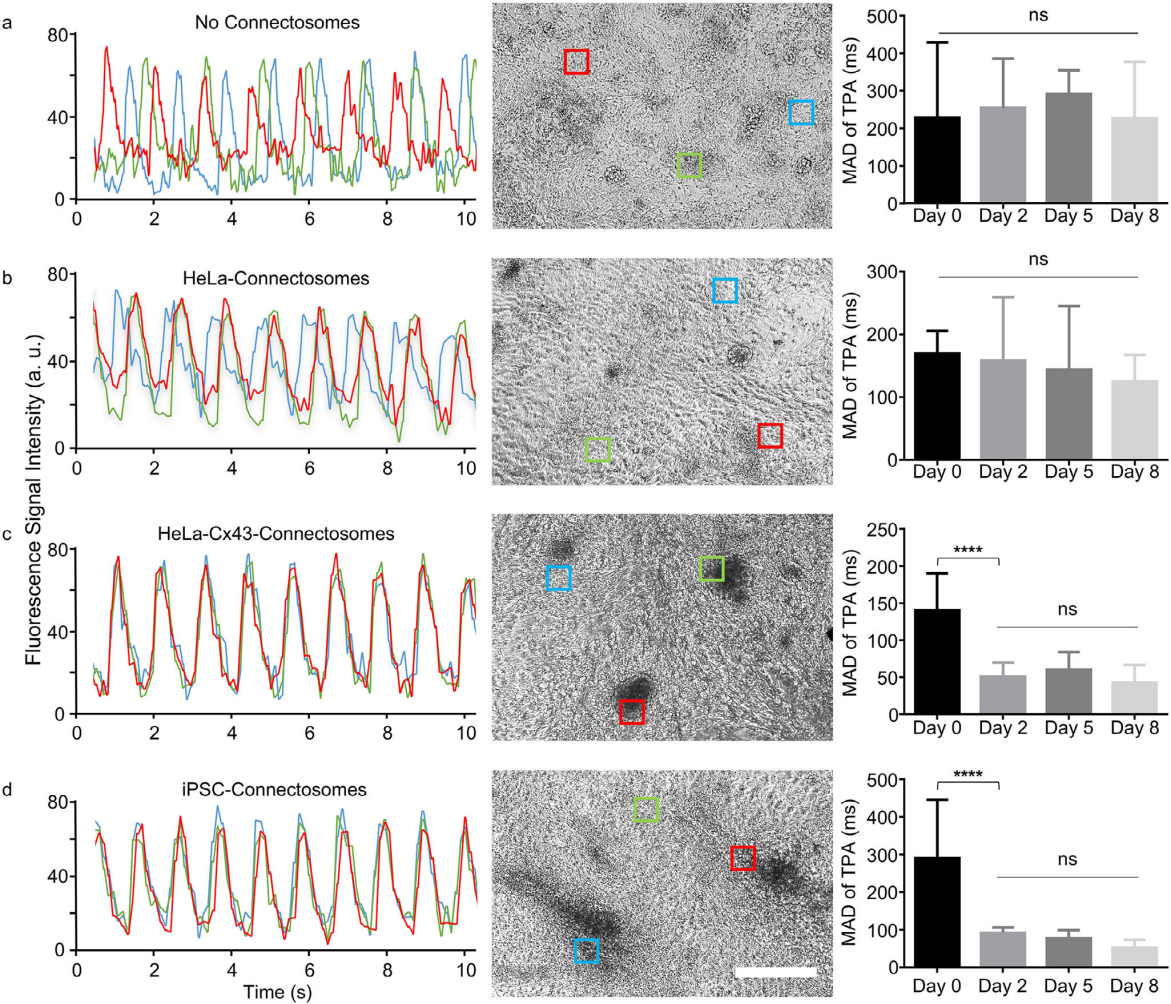
Synchronicity analysis of beating hiPSC‐CMs. (a) Negative control beating samples were not treated with Connectosomes. (b–d) Beating hiPSC‐GCaMP‐CMs were treated with membrane‐derived vesicles harvested from wild‐type HeLa cells (b), HeLa‐Cx43YFPs (c), and hiPSCs (d). Representative day 8 calcium‐transient traces (left) from three colored ROIs in the corresponding fields of view (middle) show increased synchronization following treatment with HeLa‐Cx43YFP‐ and hiPSC‐derived Connectosomes. Synchronization analysis (right) was quantified as the MAD of the TPA of Day 0 and Days 2, 5, and 8 post‐treatment. Data were collected from *n* = 6 biological replicates per condition, with 5 consecutive peaks analyzed per replicate (total 30 peaks per condition per time point). Statistical analysis was performed within each condition across time using ordinary one‐way ANOVA with Tukey's multiple comparisons; **** adjusted *p* < 0.0001; ns, not significant. All scale bars are 200 µm.

Next, to validate the computational models experimentally, we differentiated hiPSCs transfected with CMV‐GCaMP6f (hiPSC‐GCaMP) to CMs via Wnt modulation [22, 23]. The GCaMP6f is a fusion protein of green fluorescent protein and calmodulin that rapidly changes fluorescence upon binding to Ca^2^^+^ [24], allowing the tracking of calcium fluxes in CM beatings over time and quantifying changes in their beating synchronization. Synchronicity was calculated as the median absolute deviation (MAD) of the time of peak arrival (TPA) at each beating pixel cluster (Figure 5).

As predicted by our 2‐cell conductance computational model, treatment with hiPSC‐Connectosomes significantly improved the synchronicity of day 20 hiPSC‐GCaMP‐CMs beating. Calcium transients of untreated hiPSC‐ GCaMP‐CMs remained irregular and unsynchronized even after extended culture (up to 8 days; Figure 5a), indicating that synchronization was not attributable to culture time alone. As a negative control, we treated hiPSC‐GCaMP‐CMs with synthetic vesicles harvested from wild‐type HeLa cells. Because HeLa cells have little to no Cx43, these vesicles are not termed Connectosomes and did not synchronize hiPSC‐GCaMP‐CMs beating, exhibiting MAD of TPA values comparable to untreated cells (Figure 5b). To exclude potential effects of donor cell origin, we next treated hiPSC‐GCaMP‐CMs with Connectosomes harvested from HeLa cells engineered to overexpress Cx43 (HeLa‐Cx43YFP). HeLa‐Cx43YFP‐Connectosomes synchronized hiPSC‐GCaMP‐CM beating (Figure 5c) and produced a reduction in MAD of TPA comparable to hiPSC‐Connectosomes. Quantitatively, hiPSC‐Connectosomes reduced MAD of TPA by 68% within two days post‐treatment, and this trend persisted through day 8 (82% reduction vs. untreated; Figure 5d). Videos S1 and S2 show representative fields of view for hiPSC‐GCaMP‐CMs over 8 days, with hiPSC‐Connectosomes markedly improving beating synchronicity as evidenced by synchronized calcium transients (Video S1), whereas untreated cells display irregular, unsynchronized beating (Video S2). These observations suggest that Cx43 enrichment on Connectosome membranes, rather than donor cell identity, is a key determinant of improved synchronization. To assess whether Connectosome treatment broadly alters CM maturation markers or excitability, we measured expression of select markers: *PITX2* (a cardiac transcription factor linked to electrophysiologic regulation [25]) and *TNNI1*/*TNNI3* (troponin I isoforms used as sarcomere/maturation markers in hiPSC‐CMs; *TNNI1* is more fetal‐like and *TNNI3* more mature cardiac [26]) and quantified membrane potential using FluoVolt (a fast‐response voltage‐sensitive fluorescent dye for optical membrane potential measurements, Thermo Fisher). No significant differences were detected over 8 days (Figure S4), supporting the hypothesis that the primary impact of Connectosomes is mediated through Cx43‐dependent coupling rather than changes in CMs’ phenotype.

### 2.6 Connectosomes Actively Participate in Forming Connexons

**FIGURE 6:**
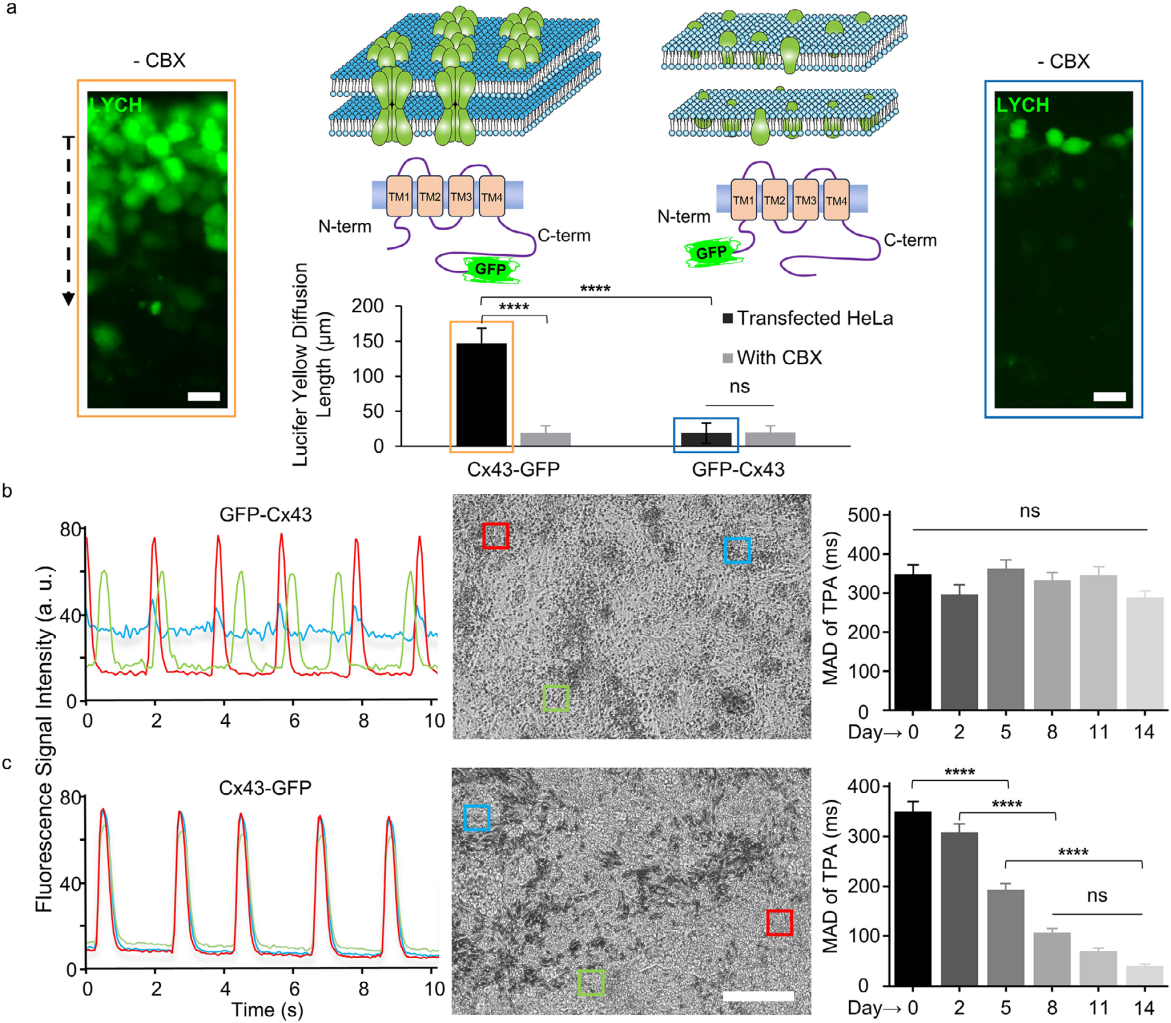
Cx43 function is sensitive to cytoplasmic domain modification. (a) Scratch assay fluorescence images showing Lucifer Yellow dye diffusion in Cx43‐GFP (left) and GFP‐Cx43 (right) transfected HeLa cells. The quantification of Lucifer Yellow diffusion length is plotted, with the schematic depicting GFP localization in different domains of Cx43. Data are mean ± SD; *n* = 8 per condition; two‐way ANOVA with multiple comparisons; significance as indicated (****p < 0.0001; ns, not significant). (b) and (c) Synchronicity analysis of calcium transients in hiPSC‐CM after treatment with Connectosomes generated from HeLa cells transfected with GFP‐Cx43 (b) and Cx43‐GFP (c) plasmids. Representative fluorescence traces from three regions of interest (colored boxes) are shown. The MAD of the TPA was measured over time, indicating variations in synchronization. Data were collected from *n* = 6 biological replicates per condition, with 5 consecutive peaks analyzed per replicate (total 30 peaks per condition per time point). Statistical analysis was performed within each condition across time using ordinary one‐way ANOVA with Tukey's multiple comparisons; **** adjusted *p* < 0.0001; ns, not significant. Scale bars: 20 µm in (a) and 200 µm in (b) and (c).

To better understand how Connectosomes reinforce the gap junction between CMs, we generated two separate reporter cell lines: HeLa cells transiently transfected with Cx43 in which GFP is conjugated to the C‐terminus (HeLa‐Cx43‐GFP) and HeLa cells transiently transfected with Cx43 in which GFP is conjugated to the N‐terminus (HeLa‐GFP‐Cx43). We chose HeLa cell lines because they have significantly low Cx43 expression; therefore, the primary source of Cx43 in these cells would be from the transfection. Transfection efficiencies were quantified by flow cytometry as the fraction of GFP‐positive cells, yielding comparable efficiencies of 53.7% for Cx43‐GFP and 46.9% for GFP‐Cx43 (Figure S5a). Prior research suggests that N‐terminal GFP tagging of Cx43 (GFP‐Cx43) can introduce steric and structural constraints that may impair formation of functional channels by affecting connexon assembly, docking, and channel gating [27]. Accordingly, we used the scratch‐loading LYCH assay as a functional readout of gap‐junctional intercellular communication to confirm the differential coupling abilities of these two cell lines. In the case of HeLa‐Cx43‐GFP cells, the average LYCH diffusion length was significantly greater than that of HeLa‐GFP‐Cx43 cells. Specifically, the LYCH diffusion length for HeLa‐Cx43‐GFP was 147.2 ± 21.5 µm, while for HeLa‐GFP‐Cx43, it was only 28.5 ± 14.4 µm, showing a 417.4% increase in diffusion length for HeLa‐Cx43‐GFP (Figure 6a). Upon exposure to CBX, both groups showed a reduction in LYCH diffusion, with HeLa‐Cx43‐GFP decreasing to 18.7 ± 10.5 µm and HeLa‐GFP‐Cx43 to 19.8 ± 9.6 µm. The decrease in LYCH diffusion for HeLa‐Cx43‐GFP under CBX was 87.3%, and for HeLa‐GFP‐Cx43, it was 30.9% (Figure 6a). Similarly, upon treatment of CMs with Connectosomes generated from HeLa‐Cx43‐GFP, we observed a similar synchronization of CM beating, as seen in Figure 5. Specifically, treatment with these Connectosomes resulted in a reduction in MAD of TPA, with a 69.5% reduction in MAD of TPA value from 0.3496 ± 0.0200 s on Day 0 to 0.1071 ± 0.0082 s on Day 8, with continued improvement at later time points (Day 11: 0.0707 ± 0.0609 s; Day 14: 0.0409 ± 0.0344 s). In contrast, when CMs were treated with HeLa‐GFP‐Cx43, we did not observe synchronization during the early time window (Days 0–8), and extending the measurements through Days 11 and 14 did not produce MAD of TPA values comparable to the HeLa‐Cx43‐GFP group (Figure 6b,c). Together, these results support that Connectosome‐mediated enhancement of cardiomyocyte synchronization depends on connexin orientation and functionality, consistent with effective intercellular coupling.

### 2.7 Connectosomes Increase Cx43 Expression in Treated Cells

We next asked whether Connectosomes impact the expression level of Cx43 within treated hiPSC‐CMs. To answer this question, we generated Connectosomes from non‐reporter hiPSCs and treated reporter hiPSC‐Cx43GFP‐CMs on Day 20 of differentiation. These experiments allowed us to track changes in Cx43 expression within the treated cells (Figure S6a). The GFP signal in hiPSC‐Cx43GFP‐CMs treated cells increased after Connectosome treatment and concentrated on the membranes of treated CMs along their junctions after Connectosome treatment. Further quantification of Cx43 expression in treated hiPSC‐CMs via flow cytometry analysis showed statistically significant 124% and 105% increases of the Median Fluorescence Intensity (MFI) in the hiPSC‐Connectosomes and HeLa‐Cx43YFP‐Connectosomes treated groups by day 8 post‐treatment (Figure S6c,d). These results suggest that the overall expression level of Cx43 increased after exposure to Connectosomes.

### 2.8 Connectosomes Increase *GJA1* Gene Expression in Treated Cells

We next asked to what extent the observed increase in Cx43 protein corresponded to changes in Cx43 transcription (*GJA1*) after Connectosome treatment. We monitored *GJA1* expression in hiPSC‐CMs at 2, 5, and 8 days post‐treatment with Connectosomes derived from non‐reporter hiPSCs. No statistically significant difference was detected through day 2, whereas *GJA1* increased by 101% and 106% on Days 5 and 8, respectively (Figure S6b). To test whether this response depends on the amount of Cx43 present in donor vesicles, we compared treatment with membrane‐derived vesicles from wild‐type HeLa cells versus HeLa‐Cx43YFP cells (comparable to hiPSC‐derived Connectosomes). In this comparison, hiPSC‐Connectosomes produced a ∼2‐fold increase in *GJA1* on Days 5 and 8, HeLa‐Cx43‐Connectosomes produced a 50% increase on Day 8, and WT HeLa vesicles produced no significant change at any time point (Figure S6b). These results demonstrate that the increase in *GJA1* expression in response to treatment requires that Connectosomes contain Cx43.

### 2.9 Connectosomes Reinforce hiPSC‐CMs' Gap Junctions

To understand the effect of Connectosomes on gap junction function between cells, we used a scratch‐loading assay to quantify dye transfer across CMs treated with Connectosomes. Specifically, we treated hiPSC‐CMs with hiPSC‐Connectosomes and then, 5 days post‐treatment, performed the scratch‐loading assay to assess gap junction continuity, as shown in Figure 1 above. We found that treating hiPSC‐CMs with hiPSC‐Connectosomes increased the LYCH diffusion length from 178.2 µm (control) to 216.5 µm (hiPSC‐Connectosome‐treated), a 21% increase (*p* = 0.0223) as shown in Figure S6e. This result indicates that treatment with Connectosomes can significantly increase LYCH permeability across layers of treated cells, demonstrating that Connectosomes strengthen the gap junction network within these cells and enhance cellular interconnectivity.

## 3 Discussion

A major driver of synchronized CM beating is electrochemical coupling [5, 6]. Electrochemical coupling is accomplished by gap junctions formed by connexins. In the adult heart, the major isoforms include connexin‐40, connexin‐43, and connexin‐45. The expression of these isoforms varies with cardiac subtype: Cx40 is primarily detected in atrial myocardium, Cx45 is associated with specialized conduction tissues, and Cx43 is most abundantly expressed in ventricular muscle. Among these connexins, Cx43 is the most abundantly expressed, whereas Cx45 is expressed at lower levels. Therefore, in this study, we focused primarily on the Cx43 isoform and its role in mediating functional coupling in iPSC‐CMs. Cx43, gap junctions are located on the plasma membrane of CMs and interconnect their cytosols [5, 28]. Immature CMs, the result of most differentiation protocols, lack extensive junction networks, which limits electrochemical coupling [29]. Previous research has shown that overexpression of Cx43 in hiPSC‐CMs increases Cx43 clustering and cluster size on their membranes and improves synchronicity [30]. Yet, this increased biosynthetic demand can strain the cell's machinery, leading to overexpression of additional proteins and impairing long‐term functionality and growth rate. In in vivo settings, virally transfected cells are at risk of mutagenesis and carcinogenesis, which limit clinical translation. Because strategies to enhance CMs’ electrochemical coupling without genetic manipulation remain limited, we investigated whether connexin‐enriched membrane‐derived vesicles (“Connectosomes”) could augment intercellular coupling and improve synchronization. There are presently few direct strategies for reinforcing gap junctions. A key advantage of this strategy is that it uses the cells’ machinery to accomplish the functional insertion of connexin transmembrane proteins into membranes. We have previously demonstrated a method for harvesting a high density of functional, properly oriented connexin hemichannels directly from the plasma membranes of donor HeLa cells, genetically engineered to express Cx43 [19]. Here, we show that Connectosomes can also be harvested from the plasma membrane of donor hiPSCs using the same mild chemical treatments that temporarily destabilize the cortical cytoskeleton [19, 31]. Once collected, hiPSC‐Connectosomes have initial diameters in the micrometer range (Figure 2). The advantage of using hiPSCs as donor cells is that they are inherently rich in Cx43. Using GFP‐Cx43 reporter hiPSCs as donor cells, we demonstrate that harvested hiPSC‐Connectosomes retain the high Cx43 expression on their surfaces and are biocompatible (Figures 2 and 3).

To assess the functionality of Cx43 hemichannels in hiPSC‐Connectosomes, we performed dye‐loading and dye‐delivery assays. We show that we can control the opening and closing of Cx43 hemichannels and release loaded dye from Connectosomes by modulating the calcium ion concentration (Figure S3). We also successfully delivered dye from the Connectosome into cells (Figure 3). Importantly, we demonstrated via modeling the conductance of two adjacent cells (Figure 4) and then experimentally on a confluent monolayer that hiPSC‐Connectosomes substantially improve the synchronization of differentiated CMs compared to untreated controls (Figure 5). A similar synchronization effect was observed when cells were treated with Connectosomes harvested from HeLa cells that were engineered to overexpress Cx43 (Figure 5). These Connectosome treatments led to a 40%–60% improvement in total synchronization, as assessed by temporal mapping of the relative time of the calcium peak across the entire field of view. Most significantly, the observed synchronization effects persisted throughout the culture period. These results demonstrate that Cx43 is the driver of Connectosomes' ability to improve synchronous beating, rather than the cell type used to produce them. Under the same culture conditions (monolayer culture without external pacing), wild‐type HeLa‐membrane‐vesicle‐treated and untreated hiPSC‐CMs frequently remained temporally heterogeneous and did not exhibit sustained field‐wide synchronization even after extended culture (up to 14 days). These results demonstrate that Connectosomes' functionality is Cx43‐dependent. We further support this conclusion by showing that Connectosomes derived from donor cells with higher Cx43‐positive fractions (engineered HeLa‐Cx43YFP) produced more robust synchronization than those generated from lower‐efficiency transient transfections (Figures 5 and 6), indicating an effective “dose” dependence on functional Cx43 availability. In this study, we used a standardized Connectosome dose (10:1 Connectosomes:cells) across all experiments to ensure direct comparisons across donor cell types. Future work will evaluate dose‐response relationships by varying the Connectosome:cell ratio and quantifying time‐to‐synchrony and additional functional endpoints.

To probe the mechanism by which Connectosomes increase synchronization, we used reporter cell lines and observed that Connectosome exposure was followed by increased *GJA1* transcription and Cx43 protein abundance of target hiPSC‐CMs (Figure S6a–d). We initially hypothesized that the Cx43‐rich surface of Connectosomes could contribute to the increased Cx43 levels in treated cells and thereby promote their synchronous beating. However, improved synchronicity was observed as early as 2 days post‐treatment, whereas increases in *GJA1* transcription and Cx43 synthesis were detected at Day 5, suggesting that the early synchronization effect precedes, and is not explained solely by the later transcriptional upregulation.

The delayed increase in *GJA1* and Cx43 transcription observed days after Connectosome treatment may reflect secondary remodeling rather than direct delivery of nucleic acids. Improved intercellular coupling can alter network activity and Ca^2^^+^‐dependent signaling, which in turn can regulate transcriptional programs associated with cardiomyocyte maturation and junctional stabilization. In addition, stabilization of junctional Cx43 at the membrane and reduced turnover could engage compensatory feedback pathways that adjust endogenous connexin expression over time [32, 33]. Additional experiments are needed to distinguish acute trafficking effects from longer‐term transcriptional regulation.

Based on these data, we conclude that the connexin‐rich surface of Connectosomes improved the stability of gap junctions. Specifically, it is established in the literature that Cx43 subunits (connexons) arrive at the plasma membrane, gradually aggregate, and assemble into gap junctions. This has been previously shown in HeLa cells; gap junction clusters grow via the migration of Cx43 subunits into the outer margins of the cluster [34]. Once junctions form, the adhesion between the two cells is so tight that connexins within the junction can be removed from the cell surface only by the formation of a double‐membrane endocytic vesicle, termed an annular gap junction. Assembly of annular gap junctions requires major rearrangements of the cytoskeleton, such that the removal of connexins within junctions is a much slower process than the removal of individual connexin pores [35]. In this way, the formation of gap junctions with neighboring cells or with a Connectosome is likely to stabilize connexins at the cell surface, thereby promoting their accumulation there. Thus, in Connectosome‐treated hiPSC‐CMs, the preferential localization of Connectosomes at cell–cell interfaces increases Cx43 accumulation at the plasma membrane of the treated cells, thereby reinforcing gap junctions and intercellular connectivity. We demonstrated increased interconnectivity via increased beating synchronization and a larger diffusive length scale in the scratch assay (Figure S6e). In particular, the scratch assay of hiPSC‐CMs treated with Connectosomes showed dye transfer over longer distances than in untreated cells. Here, Connectosomes reinforce junctions between hiPSC‐CM cells, thereby allowing the LYCH dye to pass continuously over longer distances. These data provide direct evidence that Connectosomes can reinforce junctions between hiPSC‐CM and enable interconnectivity, leading to synchronous beating.

To better understand how Connectosome reinforces gap junctions between CMs and enables their synchronized beating, we generated HeLa cell lines in which GFP expression is linked to Cx43 either through the C‑terminal (HeLa‐Cx43‑GFP) or through the N‑terminal (HeLa‐GFP‑Cx43). Prior reports demonstrate that GFP insertion at the N‐terminus sterically hinders connexon docking and gating, rendering the channels non‑functional [34]. We hypothesized that reinforcement could occur via the direct participation of Cx43 from the Connectosomes in connecting adjacent CMs, forming a new pathway for calcium flow. If this is the case, Connectosomes from the HeLa‐GFP‐Cx43 will fail to synchronize the beating of CMs. Conversely, if Connectosomes do not actively participate in connecting the cytosols of CMs but rather stabilize Cx43 at the plasma membrane, delaying its recycling, then even if connexons cannot open, Connectosomes derived from HeLa‐GFP‐Cx43 should still synchronize CM beating. Our data showed that only Cx43‑GFP Connectosomes restored cardiomyocyte synchronization, whereas GFP‑Cx43 vesicles failed to produce any beating synchronicity (Figure 6b,c), supporting that Connectosomes actively participate in connecting adjacent CMs, providing a potential approach to modulate the electrochemical coupling of CMs to each other.

These mechanistic studies further underscore the requirement for functional Cx43 hemichannels and confirm that not only is the presence of Cx43 necessary for Connectosome‑mediated coupling, but that its proper orientation and functionality are critical drivers of the observed synchronization effect.

Here, we evaluated effects in 2D hiPSC‐CM monolayers for 14 days, enabling a better understanding of the mechanistic insight into how Connectosomes enhance CM coupling and synchronization. Future studies will extend these findings by assessing durability across replating and longer culture durations, as well as performance in more mature 3D tissue models. While this work focused on Cx43 as the major driver of ventricular CM coupling, examining the roles of additional connexins and ion channels will further refine our understanding of Connectosome function. We acknowledge that a relatively simple model formulation was used to demonstrate how synchronization of calcium transients might arise between two cells. Further efforts with tighter integration between model formulation and experimental measurements will be required to shed light on the detailed mechanistic pathways. Importantly, our orthogonal experiments and controls support Connectosomes as a membrane‐derived strategy to enhance gap junction–mediated coupling and promote synchronization in hiPSC‐CM networks. Building on these results, future efforts will validate connexin orientation in treated CMs and expand functional CM analyses. Additionally, systematic profiling of Connectosomes will further optimize this platform, supporting its continued development and clinical translation.

## 4 Materials and Methods

### 4.1 Cell Lines

Human iPSC: We used three different hiPSCs cell lines: non‐reporter hiPSCs (Thermo Fisher), reporter hiPSCs expressing mEGFP‐tagged connexin‐43 (hiPSC‐Cx43GFP, Allen Institute for Cell Science), and hiPSCs transfected with CMV‐GCaMP6f (hiPSC‐GCaMP; a gift from Dr. Bruce Conklin). GCaMP6f is a fusion protein of green fluorescent protein and calmodulin that rapidly changes fluorescence upon binding to Ca^2^^+^ [24]. Human iPSCs were seeded on vitronectin‐coated 6‐well plates using Essential 8 Medium (E8, Thermo Fisher) with daily media change. Upon reaching 75% confluency, cells were passaged at a 1:20 ratio using Accutase (Innovative Cell Technologies) cell detachment solution. To improve the survival of hiPSCs after dissociation and seeding, 10 µM Y‐27632 ROCK inhibitor (Selleck Chemicals) was supplemented to the E8 media for 24 h.

HeLa cells: wild‐type, and stably transfected, inducible tet‐on HeLa cells expressing Cx43 with a C‐terminal YFP modification (HeLa‐Cx43YFP; a gift from Dr. Matthias Falk) were used [36]. HeLa cells were cultured in Dulbecco's Modified Eagle Medium (DMEM) supplemented with 10% Fetal Bovine Serum (FBS), 1% penicillin, 1% streptomycin, 1% L‐glutamine (PSLG), 100 µg/mL geneticin, and 0.4 µg/mL puromycin. To induce Cx43 expression in HeLa‐Cx43YFP, cells were incubated with 1 µg/mL doxycycline 24 h before experiments.

Experiments were performed using hiPSCs between passages P20–P40 and HeLa cells between 5–10 passages post‐thaw, and all functional assays were replicated across independent Connectosome preparations.

### 4.2 Generation of HeLa‐GFP‐Cx43 and HeLa‐Cx43‐GFP Cell Lines

HeLa cells maintained in growth medium (DMEM/F12 supplemented with 10% fetal bovine serum (FBS, Corning 35‐017‐CV) and 1% antibiotic‐antimycotic (anti‐anti, Sigma Aldrich A5955)) were passaged when 70%–80% confluent at a 1:10 split into tissue culture‐treated 6‐well plates and transfected 48 h later. Cx43‐GFP and GFP‐Cx43 transfection solutions were prepared by combining 1000 ng of plasmid with 3 µL FuGENE HD (Promega E2311) and DMEM/F12 (serum and antibiotic‐free) to a total volume of 100 µL in a 1.5 mL microcentrifuge tube, with 100 µL prepared per well, undergoing transfection. The solution was inverted to mix and incubated at room temperature for 15 min. After incubation, the medium in each well was replaced with 900 µL of growth medium, and 100 µL of Cx43‐GFP and GFP‐Cx43 transfection solution was added dropwise to their respective wells. HeLa cells were transfected in 100 mm Petri dishes using the same process, with an amount of plasmid DNA and volumes of solutions multiplied by a factor of 8.25. The transfection medium was exchanged with the growth medium about 18 h later. Forty‐eight hours later, transfected cells were either utilized for generating Connectosomes or detached for flow cytometry to assess transfection efficiency based on the GFP fluorescent signal. Flow cytometry was performed on a Sony MA900 Multi‐Application Cell Sorter (Center for Biomedical Research Support Microscopy and Imaging Facility at UT Austin. RRID:SCR_021756), with ≥30 000 total events recorded per sample.

### 4.3 Cell Conductance Modeling

We developed a computational model to simulate the dynamics of membrane potential at the plasma membrane and calcium concentrations across various subcellular compartments in two adjacent cells. The purpose of this model is to provide a mechanistic link between the hypothesized primary effect of Connectosome treatment, which is an increase in effective intercellular coupling, and the expected reduction in calcium transient timing dispersion. Connectosome treatment is implemented by increasing the gap‐junction coupling parameter *g* relative to the untreated condition. The model is used to evaluate qualitative effects of increased coupling on synchronization and is not fit to estimate absolute intracellular calcium concentrations from the experimental data. Our model was adapted from a previous study [21], where the membrane potential is governed by the applied stimulus current and ionic currents through various channels, including sodium, potassium, and calcium channels, as well as currents mediated by calcium and sodium‐potassium pumps. The original model also accounts for calcium dynamics within several compartments, including the dyad, bulk cytosol, subsarcolemmal space (SL), junctional sarcoplasmic reticulum, and network sarcoplasmic reticulum. However, because the model developed by Jaeger et al. [21] does not incorporate gap junction coupling, we introduced an additional term in the membrane potential equation to represent intercellular electrical coupling through gap junctions. We refer the reader to Jaeger et al. [21] for more details, and here we only listed the equations for membrane potential *V_m_* and cytosolic calcium concentration *c_c_*:

$$\;\frac{dV_{m}^{\left(1\right)}}{dt}=-I_{ion}^{\left(1\right)}-g\left(I_{gap}^{\left(1\right)}-I_{gap}^{\left(2\right)}\right)$$

$$\;\frac{dV_{m}^{\left(2\right)}}{dt}=-I_{ion}^{\left(2\right)}-g\left(I_{gap}^{\left(2\right)}-I_{gap}^{\left(1\right)}\right)$$

$$\;\frac{dc_{c}^{\left(i\right)}}{dt}=\frac{1}{V_{c}}\;\left(J_{sl}^{c,\left(i\right)}+J_{d}^{c,\left(i\right)}-J_{c}^{n,\left(i\right)}-J_{c}^{b,\left(i\right)}\right),\;i=1,2$$

The superscripts 1 and 2 correspond to cells 1 and 2, respectively. In the equations for *V_m_*, $I_{ion}^{\left(1\right)}$ and $I_{ion}^{\left(2\right)}$ are the sum of ionic currents through the membrane of cells 1 and 2, respectively. Here, $I_{ion}^{\left(1\right)}$ and $I_{ion}^{\left(2\right)}$ have the same form for both cells, which is the summation of membrane currents *I*_Na_, *I*_NaL_, *I*_CaL_, *I*_to_, *I*_Kr_, *I*_Ks_, *I*_K1_, *I*_NaCa_, *I*_NaK_, *I*_pCa_, *I*_bCl_, *I*_bCa_, *I*_f_, and the applied stimulus *I*_stim_. The $g(I_{gap}^{\left(1\right)}-I_{gap}^{\left(2\right)})$ and $g(I_{gap}^{\left(2\right)}-I_{gap}^{\left(1\right)})$ are the newly added terms that are used to model the gap junctions, where *g* is the gap junction conductance and the $I_{gap}^{\left(i\right)},\;i=1,2$ is written as follows:

$$\;I_{gap}^{\left(i\right)}=V_{m}^{\left(i\right)}\;-\frac{RT}{F}\log\left(\frac{{\left[Ca^{2+}\right]}_{e}}{c_{c}^{\left(i\right)}}\right)$$

where *F* is Faraday's constant. *R* is universal gas constant; *T* is temperature and [*Ca* ^2 +^]_*e*_ is the extracellular calcium concentration. In the equations for *c_c_*, $J_{sl}^{c}$, $J_{d}^{c}$, $J_{c}^{n}$, and $J_{c}^{b}$ are fluxes among different compartments. The equations for *V_m_* and *c_c_* are coupled in the following ways: *I*_CaL_, *I*_NaCa_, *I*_pCa_, *I*_bCa_, *I*_gap_ are affected by the intracellular calcium concentrations; the *c_c_* is affected by the flux between SL and bulk cytosol $J_{sl}^{c,(i)}$, and the calcium fluxes in the SL are affected by calcium channels and pumps on the cell membrane. For more details, we refer the readers to Jaeger et al. [21].

Kinetic parameters were directly taken from Jaeger et al. and are the same for both cells except for the applied stimulus current. The applied stimulus current *I*_stim_ is zero for most of the time. However, cell 1 receives a − 40 mV applied stimulus during the interval [100 + 1000*N*, 101 + 1000*N*] ms, and the cell 2 during [250 + 1000*N*, 251 + 1000*N*] ms, where N=1,2,3,… Furthermore, since the treatment with hiPSC‐Connectosomes leads to a high density of connexin hemichannels, we assumed that the gap junction conductance *g* also increases. Therefore, we chose *g*  =  0 for the case without the treatment of hiPSC‐Connectosomes and *g*  =  0.1 for the case with the treatment. The model code is available at https://github.com/RangamaniLabUCSD/Connectosome_model.

### 4.4 Cardiomyocyte Differentiation

We employed the Wnt GiWi method [22, 23] to differentiate hiPSC to CMs. Briefly, hiPSCs were detached using Accutase and seeded at 25 000 cells/cm^2^ in 6‐well cell culture plates coated with Matrigel (Corning) in E8 media supplemented with 10 µM ROCK inhibitor for 24 h. The cells were expanded in E8 for an additional 2 days, reaching approximately 85% confluency. On Day 0 of differentiation, hiPSCs were treated with 12 µM of CHIR99021 (LC Laboratories) in RPMI 1640 supplemented with B27 without insulin (RB‐, Life Technologies). On Day 1, CHIR99021 was removed, and cells were cultured in RB‐media for an additional 48 h. On day 3 of differentiation, 50% of the media was replaced with fresh RPMI 1640 (Tocris), and IWP‐2 (Tocris) was added to a final concentration of 5 µM. On day 5, IWP was removed, and cells were cultured in RB‐media. After 48 h, RB‐ media was replaced with RPMI 1640 supplemented with B27 with insulin (RB+, Life Technologies). On Day 8, the media was changed to RPMI 1640 with no glucose (Thermo Fisher), supplemented with B27 (Life Technologies). After 24 h, the media was changed back to RB+ and renewed every 3 days thereafter.

### 4.5 Connectosome Formulation

Connectosomes were harvested directly from the plasma membrane using an osmotic swelling step to favor membrane blebbing, followed by incubation in a Ca^2^^+^‐containing vesiculation buffer with mild vesiculation agents that transiently weaken membrane‐cortex attachment [31, 37, 38, 39]. Donor cells for the extraction of Connectosomes, including reporter hiPSC‐Cx43GFPs, HeLa‐Cx43YFPs, and wild‐type HeLa cells, were induced to form Connectosomes or giant plasma membrane vesicles (GPMVs) using an established protocol [16, 17]. Briefly, upon reaching 95% confluence, media was removed, and cells were then rinsed and incubated with active GPMV buffer (10 mM HEPES, pH 7.4, 2 mM CaCl_2_, 150 mM NaCl, 2 mM N‐ethylmaleimide) at 37°C for 8 h. The active GPMV buffer containing Connectosomes was then collected and centrifuged at 250 xg for 5 min to remove the cell debris. Then, the supernatant was centrifuged at 17 000 xg at 4°C for 15 min. The supernatant was removed, and the Connectosomes were resuspended in DPBS.

### 4.6 Connectosome Characterization

The Connectosomes were characterized for their size and number using a Scepter 2.0 (MilliporeSigma) handheld automated cell counter. We also assessed the cytotoxicity of Connectosomes using the LIVE/DEAD Viability Cytotoxicity Kit (Thermo Fisher) according to the manufacturer's procedure. Briefly, non‐reporter hiPSCs (Thermo Fisher) were seeded in 48‐well plates and allowed to reach 85% confluency. Connectosomes were then suspended in E8 media (at 10 times the number of cells in each well) and incubated with hiPSCs at 37°C for 24 h. Next, cells were incubated in PBS containing 2 µM Calcein acetomethoxy (AM) and 4 µM ethidium homodimer‐1 at 37°C for 30 min. The fluorescence signals of live and dead cells were then measured at 530 nm and 645 nm wavelengths, respectively, using a Synergy (BioTek) microplate reader.

### 4.7 Scratch Dye Loading Assay

hiPSCs were seeded on vitronectin‐coated glass coverslips in 6‐well plates at a density of 100 000 cells per well. Cells were cultured for 5 days, allowing them to reach 95% confluency. The coverslips were rinsed three times with PBS and immersed in PBS containing 0.25% LYCH dye (Sigma‐Aldrich). A 2 µL micropipette tip was loaded with LYCH and used to create a longitudinal scratch through the monolayer, releasing the dye at the same time. The cells were incubated for 7 min at 37°C and then rinsed with PBS three times. The same experiments were repeated in the presence of a gap junction blocker, Carbenoxolone (CBX). Briefly, cells were incubated in PBS containing 100 µM Carbenoxolone (CBX) at 37°C for 30 min before making the scratch. The cells were immersed in 100 µM CBX as the scratch was performed, and were rinsed three times with PBS containing 100 µM CBX. LYCH fluorescence was visualized under the spinning disc confocal microscope with a 405 nm laser and a 525 nm filter at 50 nm width.

We also performed the scratch dye loading assay on Day 20 non‐reporter hiPSC‐CMs treated with Connectosomes generated from non‐reporter hiPSCs. Connectosomes were added to the beating hiPSC‐CMs in RB+ media at a ratio of 10:1 Connectosomes to cells. RB+ with no Connectosome was used as the negative control. After 5 days, the media was removed, the cells were rinsed with PBS, and a longitudinal scratch was made using a 2 µL micropipette tip loaded with LYCH, releasing the dye simultaneously. The cells were then incubated for 7 min at 37°C, rinsed with PBS three times, and the fluorescence was visualized using a confocal microscope.

### 4.8 Western Blot Analysis

To assess Cx43 protein abundance in donor cell lines (hiPSCs and HeLa‐Cx43‐YFP), cells were washed with ice‐cold Dulbecco's Phosphate Buffered Saline (DPBS) and lysed on ice in 1% NP‐40 lysis buffer (100 mM Tris, pH 7.5; 100 mM NaCl; 1% NP‐40). Lysates were clarified by centrifugation at 4°C, mixed with 4× SDS–PAGE loading buffer containing a reducing agent, and resolved on a 4%–12% Bolt Bis‐Tris Plus polyacrylamide gel. Proteins were transferred to a nitrocellulose membrane, blocked for 1 h at room temperature in LI‐COR blocking buffer, and probed with primary antibodies against Cx43 (Thermo Fisher, 1:1000) and GAPDH (Thermo Fisher, 1:1000) diluted in 1× TNET wash buffer (10 mM Tris, pH 7.5; 2.5 mM EDTA; 50 mM NaCl; 0.1% Tween‐20). After three washes in TNET (5 min each), membranes were incubated for 1 h at room temperature with Goat anti‐Mouse IgG Alexa Fluor 680 secondary antibody (Thermo Fisher, 1:10 000) in antibody diluent, washed again (3×, 5 min), and imaged on a LI‐COR Odyssey Imaging system.

### 4.9 Connectosome Dye Loading, Release, and Transfer

A 2 mg/mL stock solution of CRO dye in DMSO was prepared and diluted to the final concentration of 20 ng/µL in PBS. hiPSC‐Cx43GFP cells were incubated in the CRO AM dye solution for 30 min and were used to form CRO dye‐loaded Connectosomes. Non‐reporter hiPSCs were seeded on vitronectin‐coated glass coverslips in 6‐well plates at 100,000 cells per well. After 24 h, CRO dye‐loaded Connectosomes were added to the cells at a ratio of 10:1. The cells were imaged immediately on the spinning disk confocal microscope to illustrate the transfer of CRO dye from the Connectosomes into the non‐reporter hiPSCs.

Additionally, we used CRO‐loaded Connectosomes to demonstrate the functionality of the connexin channels on the Connectosomes’ membrane by modulating the opening and closing of the connexin and measuring Cro dye release. Donor cells were incubated with CRO AM dye solution for 30 min. The CRO‐labeled cells were then rinsed with GPMV buffer to remove the excess dye and incubated with active buffer at 37°C for 8 h. Connectosomes were then isolated and resuspended in GPMV buffer containing 2 mM Ca^2^^+^. 5 mM EGTA and EDTA chelators were then added to remove the calcium and open the channels. Connectosomes were immediately monitored for dye release using a Zeiss AxioObserver confocal microscope with Yokagawa Spinning Disk, equipped with a 100x oil immersion (NA 1.4) objective and an EMCCD iXon3 897 (Andor Technology) camera.

### 4.10 Gene Expression

We analyzed the gene expression profile on day 20 differentiated non‐reporter hiPSC‐CMs following treatment with Connectosomes. CMs were cultured in 6‐well plates and treated with Connectosomes (*n* = 3 per condition). At set time points following incubation with Connectosomes, cells were rinsed twice with DPBS, and the total mRNA was isolated using RNeasy Mini Kit (QIAGEN), followed by reverse transcription using the High‐Capacity cDNA Reverse Transcription Kit (Thermo Fisher) according to manufacturer's protocols. RT‐qPCR was performed with PowerUp SYBR green (Thermo Fisher) using the StepOnePlus Real‐Time PCR System (Applied Biosystems). 25 ng of cDNA and 500 nM primers were used for each reaction. Reactions were activated at 50°C for 2 min and then at 95°C for 2 min. Forty amplification cycles (95°C for 15 s, followed by annealing at 60°C for 60 s) were performed. Relative expression of mRNA was quantified using the ∆∆CT method with Human GAPD (GAPDH) as the endogenous control and mRNA from non‐treated Day 20 CMs as the reference. *GJA1* (Bio‐Rad), cardiac troponin I (TNNI3), slow skeletal troponin I (TNNI1), and pituitary homeobox 1 (PITX2) human primers were used to analyze the specific gene expression.

### 4.11 Cytometry

All cell types used for flow cytometry were cultured as adherent monolayers. Immediately prior to detachment, culture medium containing floating cells was aspirated, and cells were rinsed with DPBS to remove detached and dead cells. Quantitative assessment of Cx43 protein production in hiPSC‐CMs was performed by flow cytometry analysis of reporter hiPSC‐Cx43GFP‐CMs. Connectosomes were harvested from three donor cell types: non‐reporter hiPSC, wild‐type HeLa, and HeLa‐Cx43YFP, which were resuspended in RB+ (or E8 for dye delivery) and incubated with hiPSC‐Cx43GFP‐CMs (or hiPSCs). For the CRO dye delivery, hiPSCs after 24 h or for the Cx43 synthesis analysis, hiPSC‐Cx43GFP‐CMs after 2, 5, and 8 days, were rinsed with DPBS and dissociated with Accutase. A BD Accuri C6 Plus system was used to collect the data with 100 000 counts of each sample. Using FlowJo v10 software, debris and doublets were excluded using FSC‐A vs FSC‐H gating, and analysis was performed on the singlet population. The histograms were then used to measure the geometric median cell fluorescence intensities (MFI) to compare different treatment groups at separate time points.

### 4.12 Optical Membrane Potential Measurements (FluoVolt)

Day 20 hiPSC‐CMs were treated with hiPSC‐Connectosomes (10:1 Connectosomes:cells) or left untreated. At Days 0, 2, 5, and 8 post‐treatment, membrane potential was assessed using FluoVolt (Thermo Fisher) according to the manufacturer's protocol. Briefly, cells were incubated with FluoVolt dye in assay buffer and then equilibrated at room temperature prior to fluorescence recording. FluoVolt fluorescence was measured using a BioTek Synergy plate reader under identical acquisition settings for all groups and time points, and the mean fluorescence intensity for each well was recorded. FluoVolt fluorescence was used as a relative reporter of membrane potential and was not calibrated to absolute voltage.

### 4.13 Synchronicity Analysis of Connectosome‐Treated hiPSC‐GCaMP‐CMs

Day 20 hiPSC‐GCaMP‐CMs were used to analyze the impact of Connectosomes on the synchronicity of their beating. Connectosomes were freshly prepared for each experiment, harvested from non‐reporter hiPSCs, HeLa‐Cx43YFPs, and wild‐type HeLa cells, and suspended in RB+. Connectosomes and a sample well of hiPSC‐GCaMP‐CMs were counted using a Scepter 2.0 (MilliporeSigma) handheld automated cell counter. Connectosomes were then added to hiPSC‐GCaMP‐CMs in RB+ media at a ratio of 10:1 Connectosomes to cells. The same dose was used across donor types. RB+ media with no Connectosome was added to hiPSC‐GCaMP‐CMs, serving as negative controls. From each well, 5 fields of view (FOVs) with beating cells were selected on day 0. The precise locations of these FOVs were recorded, and distinct morphological features within each FOV (e.g., cell cluster shapes, debris patterns, or micro‐scratches) were used as landmarks. These same FOVs were then revisited and re‐imaged on Days 2, 5, and 8. Brightfield and green fluorescence 1‐minute videos of each FOV were recorded on Days 0 (before adding the Connectosomes), 2, 5, and 8, and the synchronicity of contracting cells was quantified. Videos were captured at 60 frames/s using an EVOS FL/FL Color microscope (Thermo Fisher) connected to a Windows 10 machine running Open Broadcaster Software (OBS) with a USB 3.0 video capture card. Green channel fluorescence videos from beating hiPSC‐GCaMP‐CMs were recorded in uncompressed AVI format. From each FOV video file, 3 to 5 identically sized regions of interest (ROIs) were selected as clusters of pixels to collectively span the beating region of the field FOV and mask out the non‐beating areas. The average fluorescence intensity in each ROI was measured in each frame using ImageJ (NIH) software. A chart of fluorescence intensity versus time was created for each ROI, and the synchronicity was quantified as the MAD of the TPA across all of the ROIs within each FOV. GCaMP6f fluorescence was used as a relative reporter of calcium transients for timing/synchrony analysis (peak timing), and signals were not calibrated to absolute intracellular Ca^2^^+^.

### 4.14 Statistical Analysis

Statistical analyses were performed in GraphPad Prism 6 (GraphPad). Data are presented as mean ± SD unless otherwise indicated, and n represents independent biological replicates as defined for each experiment in the corresponding figure legend. Statistical tests were chosen based on the experimental design (e.g., unpaired two‐tailed t test for two‐group comparisons; one‐way ANOVA with Tukey's multiple‐comparisons test for ≥3 groups; two‐way ANOVA with Šidák's multiple‐comparisons test for two‐factor designs), with nonparametric alternatives used when assumptions for parametric testing were not met. All tests were two‐sided with α = 0.05. Exact tests, multiple‐comparisons corrections, and *p‐*values are reported in the figure legends.

## Author Contributions

Conceptualization: Janet Zoldan and Jeanne C. Stachowiak. Methodology: Nima Momtahan, Jeanne C. Stachowiak, and Janet Zoldan. Investigation: Nima Momtahan, Anna K. McClain, Andrea Trementozzi, Sogu Sohn, Chi Zhao, Lingxia Qiao, and Padmini Rangamani. Visualization: Nima Momtahan, Anna K. McClain, Andrea Trementozzi, Sogu Sohn, Chi Zhao, Lingxia Qiao, and Padmini Rangamani. Supervision: Janet Zoldan and Jeanne C. Stachowiak. Writing – original draft: Nima Momtahan, Janet Zoldan, Anna K. McClain, Andrea Trementozzi, Sogu Sohn, and Jeanne C. Stachowiak. Writing – review & editing: Janet Zoldan, Nima Momtahan, and Jeanne C. Stachowiak.

## Conflicts of Interest

The authors declare no conflicts of interest.

## Funding

National Science Foundation (2002652, J.Z.), Texas Biologics (TXB‐24‐04, J.Z.), National Science Foundation (2530428, J.C.S.), National Science Foundation (2228270, J.C.S.), National Institute of Health (R35GM139531, J.C.S.), Welch Foundation (F‐2257, J.C.S.), Army Research Office (W911NF2310249, P.R.).

## Supporting information




**Supporting File 1**: advs75032‐sup‐0001‐SuppMat.docx.

**Supporting File 2**: advs75032‐sup‐0002‐VideoS1.mp4.

**Supporting File 3**: advs75032‐sup‐0003‐VideoS2.mp4.

## Data Availability

The data that support the findings of this study are available from the corresponding author upon reasonable request.
