# Supplementary material for: Connexin 43‐Enriched Vesicles Improve Synchronization in hiPSC‐Derived Cardiomyocytes
Source: Adv Sci (Weinh). 2026 Apr 28;13(33):e21032. doi: 10.1002/advs.202521032 (PMC13271624; doi:10.1002/advs.202521032)
Supplement: Supplementary file 1 — Supporting File 1: advs75032‐sup‐0001‐SuppMat.docx. [file ADVS-13-e21032-s002.docx]

## Figures

## Supplementary Materials


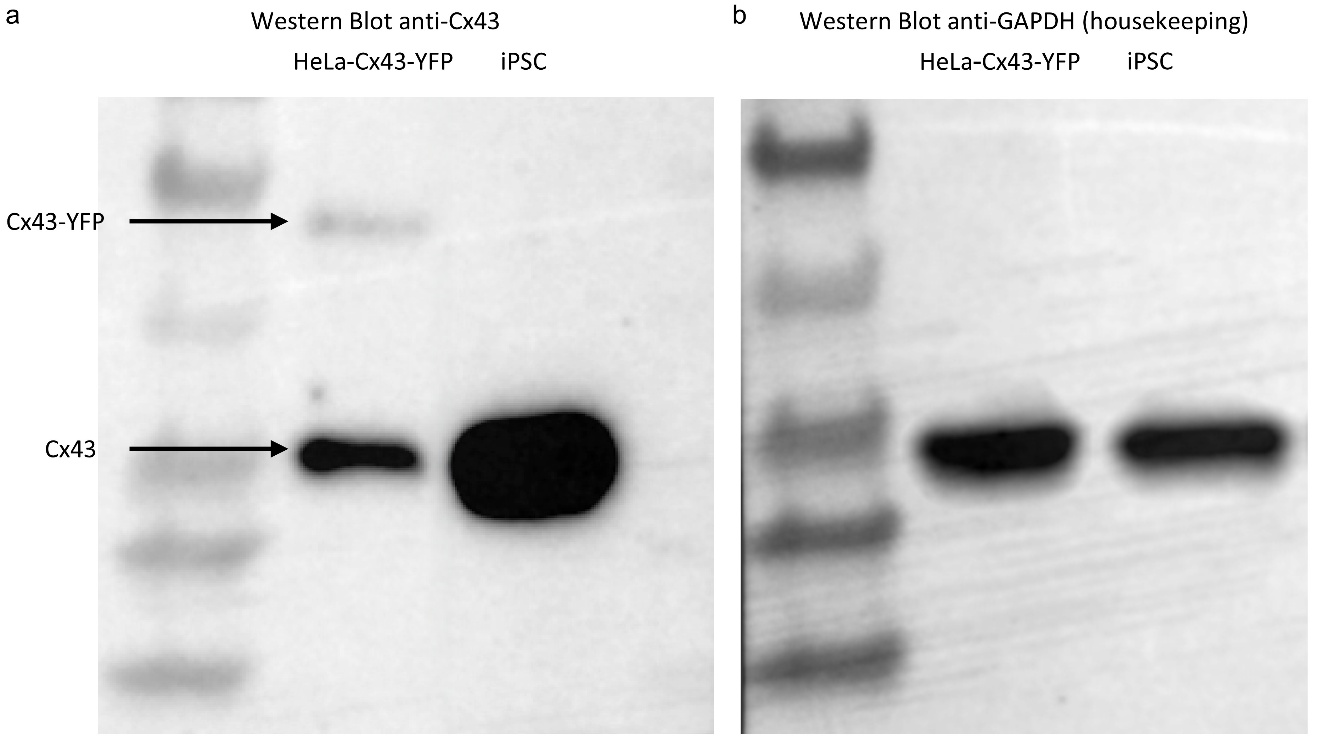


**Figure S1.** **Connexin 43 protein expression in donor cell lines by immunoblotting.** (a) Representative Western blot probed with anti-Cx43 comparing HeLa–Cx43–YFP and hiPSC lysates. Bands corresponding to endogenous Cx43 and the higher–molecular weight Cx43–YFP fusion protein are indicated. (b) The same lysates were probed with anti-GAPDH as a housekeeping/loading control.


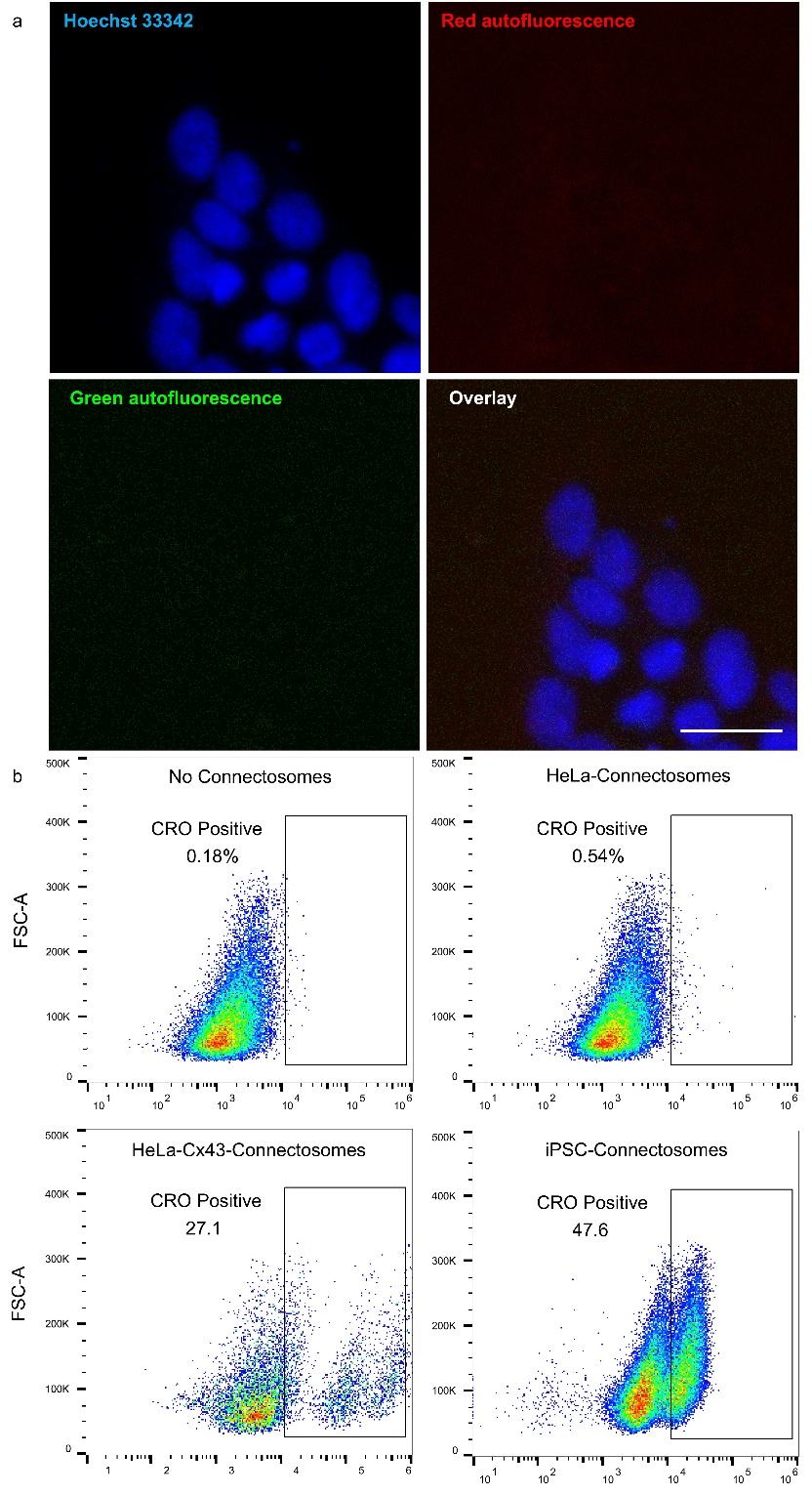


**Figure S2. Autofluorescence control and flow-cytometry quantification of CRO dye delivery to hiPSCs.**
(a) Representative fluorescence images of untreated hiPSCs (no Connectosomes) showing Hoechst 33342 nuclear staining (blue) and baseline signal in the red and green channels used for CRO detection, demonstrating minimal cellular autofluorescence under the imaging conditions. The overlay image shows Hoechst with red/green channels. Scale bar: 10 µm.
(b) Representative flow cytometry analysis of CRO-positive hiPSCs 24 h after treatment with Connectosomes loaded with calcein red-orange (CRO). Representative dot plots and gates are shown for no Connectosomes (0.18% CRO-positive), regular HeLa-Connectosomes (0.54% CRO-positive), HeLa-Cx43-Connectosomes (27.1% CRO-positive), and hiPSC-Connectosomes (37.6% CRO-positive), indicating enhanced CRO delivery with Cx43-enriched Connectosomes.

**
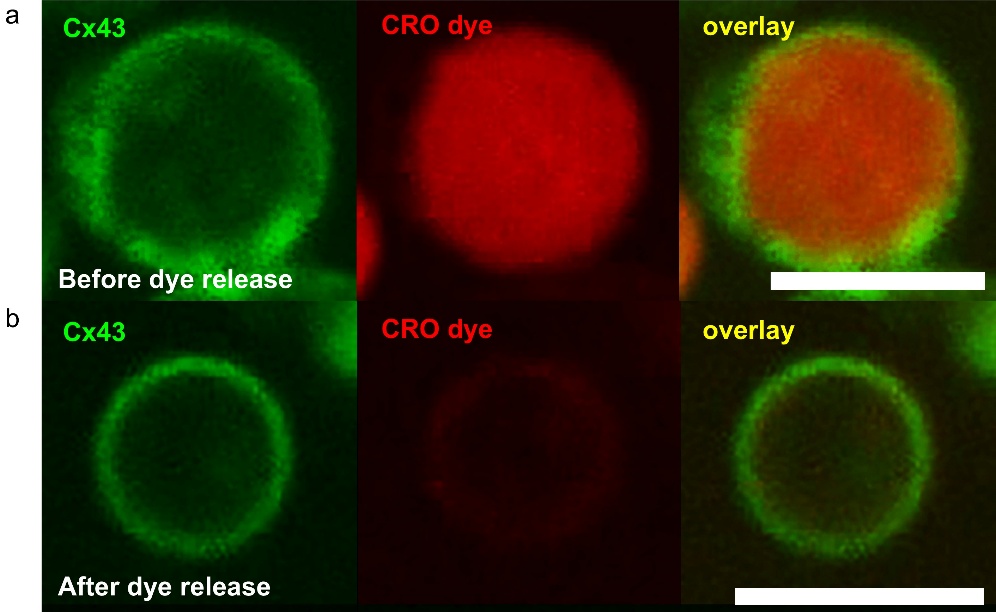
**

**Figure S3.** **CRO dye release from Connectosomes. Representative fluorescence images of Connectosomes from hiPSC-Cx43GFPs stained with CRO.** (a) Before dye release: Connectosomes were maintained in GPMV buffer containing 2 mM Ca²⁺ to keep hemichannels closed and retain CRO within the vesicle lumen. Green: Cx43-GFP membrane; red: CRO; overlay shown at right. (b) After dye release: Ca²⁺ was removed by addition of EDTA/EGTA (5 mM) to chelate Ca²⁺, a condition expected to promote connexin hemichannels opening, resulting in decreased vesicular CRO signal consistent with dye release in this endpoint proof-of-principle assay. Green: Cx43-GFP membrane; red: CRO; overlay shown at right. Scale bars: 5 µm.


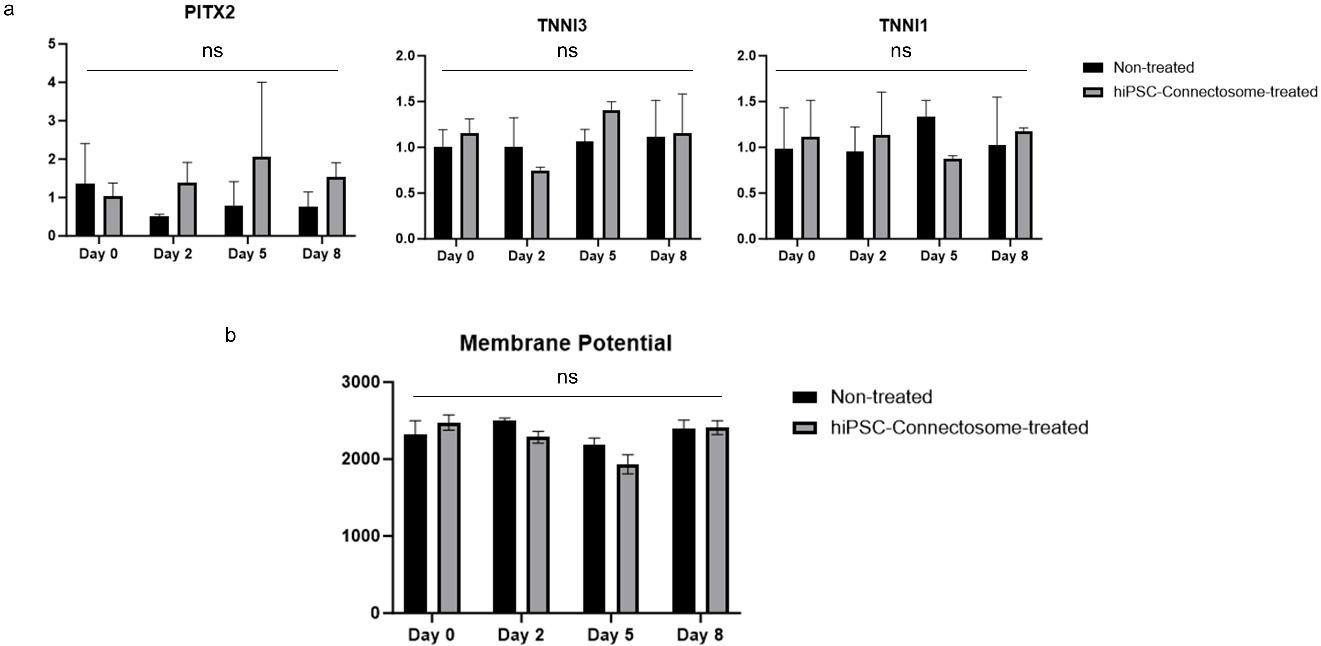


**Figure S4. Connectosome treatment does not significantly alter selected cardiac gene expression markers or membrane potential over 8 days.**
(a) RT-qPCR analysis of PITX2, TNNI3, and TNNI1 expression in hiPSC-CMs that were untreated or treated with hiPSC-Connectosomes and assessed at Day 0, 2, 5, and 8 post-treatment. (b) Membrane potential measurements of untreated and hiPSC-Connectosome–treated hiPSC-CMs using FluoVolt (Thermo Fisher) at Day 0, 2, 5, and 8. Data are presented as mean ± SD. n = 4. Statistical analysis: two-way ANOVA (or two-way RM ANOVA where applicable) testing effects of time, treatment, and their interaction; ns, not significant.


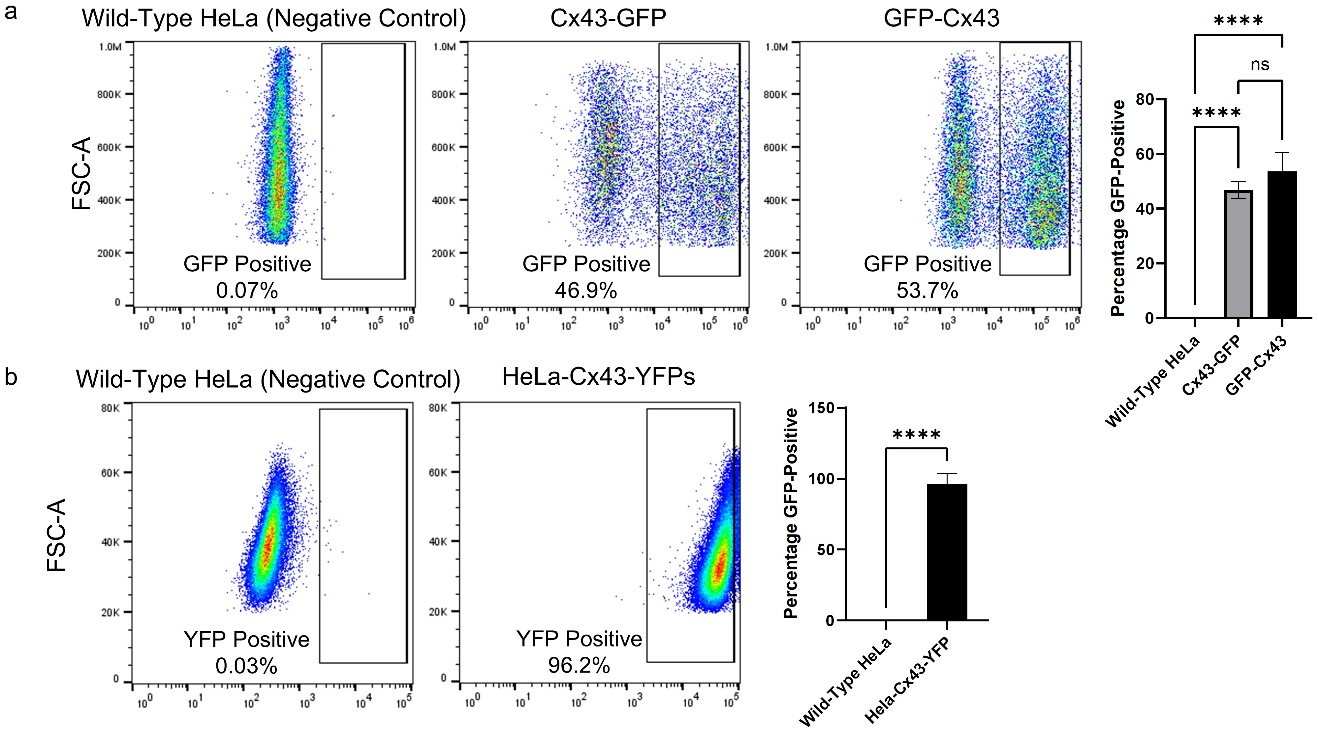


**Figure S5. Flow cytometry quantification of donor-cell reporter expression and transfection efficiency.** (a) Representative flow-cytometry plots of GFP positivity in wild-type HeLa (negative control) and transiently transfected HeLa expressing Cx43–GFP or GFP–Cx43, with quantification. (b) Representative flow-cytometry plots of YFP positivity in regular HeLa (negative control) and engineered HeLa–Cx43–YFP cells, with quantification.

Statistical analysis: (a) n = 3; ordinary one-way ANOVA with Šidák’s multiple comparisons (**** adjusted *p* < 0.0001; ns, not significant). (b), n = 3; unpaired two-tailed t-test (**** *p* < 0.0001).


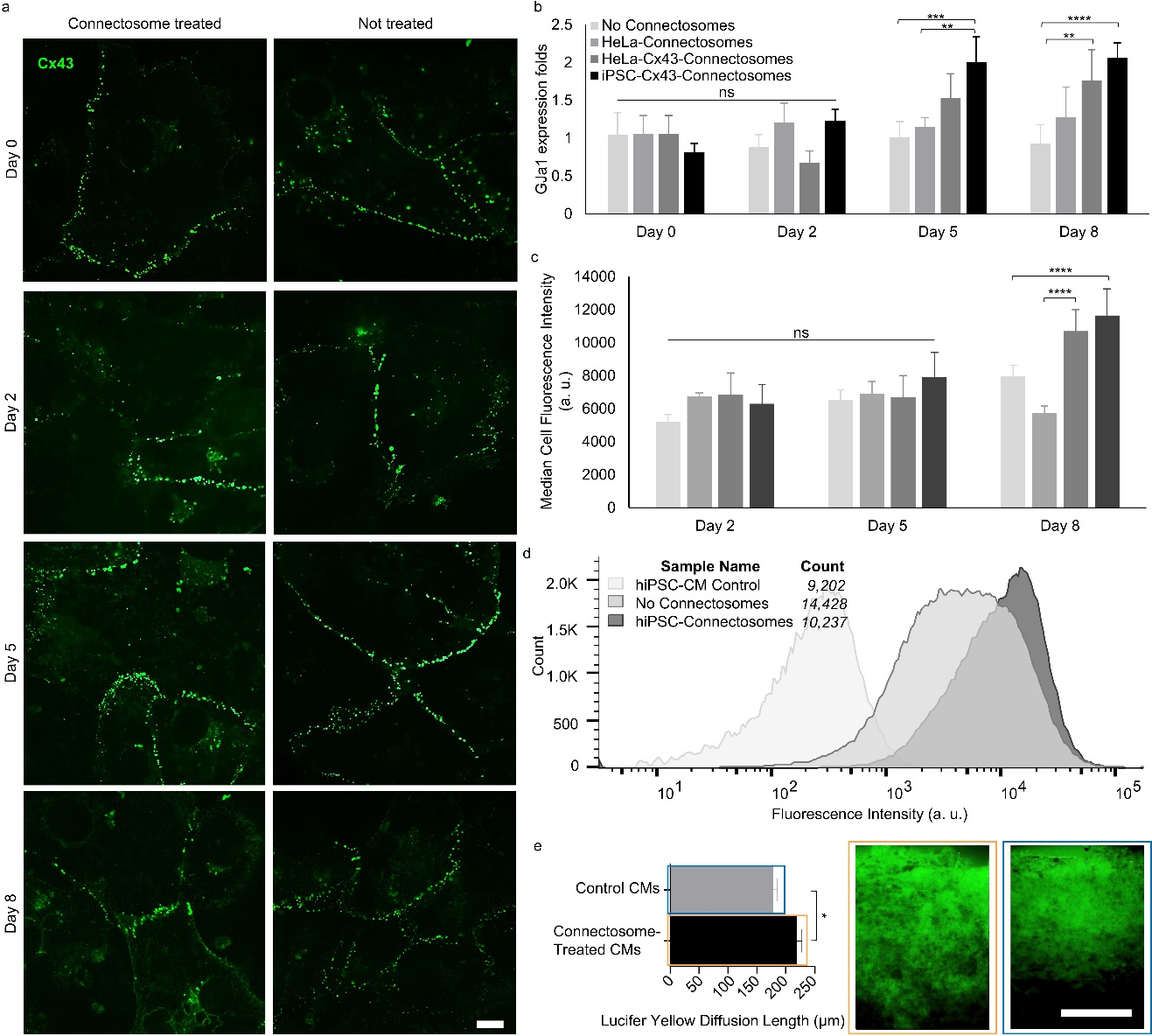


**Figure S6. Fluorescence imaging, RT-qPCR gene expression, and flow cytometry analysis of Cardiomyocytes differentiated from Cx43-reporter-hiPSCs (hiPSC-Cx43GFP-CMs) treated with Connectosomes harvested from non-reporter hiPSCs.** (a) Cx43-GFP reporter fluorescence images of cardiomyocytes before, 2, 5, and 8 days after iPSC-Connectosome treatment show a qualitative increase of green fluorescence signal in the Connectosome-treated group. (b) RT-qPCR analysis of cardiomyocytes mRNA shows a statistically significant increase in the expression of *GJA1* in the HeLa-Cx43-Connectosome and iPSC-Connectosome treated cardiomyocytes after 5 days compared to non-treated. (c) Flow cytometry quantitative analysis of hiPSC-Cx43GFP-CMs shows a statistically significant increase in the median fluorescence intensity of cardiomyocytes 8 days after treatment with HeLa-Cx43-Connectosomes and iPSC-Connectosomes. (d) The right shift of the Count vs. Fluorescence Intensity chart of cardiomyocytes 8 days after treatment, iPSC-Connectosomes. (e) The diffusion distance of LYCH dye in hiPSCs-Connectosomes-treated CMs compared to non-treated CMs (control). Statistical analysis: Data are mean ± SD; for (b–c): n=5, ordinary one-way ANOVA with Tukey’s multiple comparisons; **=P value ≤ 0.01, ***=P value ≤ 0.001, ****=P value ≤ 0.0001, ns, not significant; for (e): n = 5 per group. Statistical analysis: two-tailed unpaired t-test (p = 0.0223). *=p < 0.05). Scale bars: 10 µm in (a) and 100 µm in (e).

**Video S1: Time-lapse of hiPSC-GCaMP-CMs treated with iPSC-Connectosomes, showing synchronized beating over 8 days.** The right column displays the green fluorescence channel (GCaMP GFP signal) used to analyze beating synchronizations, while the left column shows the corresponding brightfield images of the same area.

**Video S2: Time-lapse of hiPSC-GCaMP-CMs without treatment, showing unsynchronized beating over 8 days.** The right column displays the green fluorescence channel (GCaMP GFP signal), while the left column shows the corresponding brightfield images of the same area.
